# An essential gene screening identifies yeast Mot1 as a suppressor of R-loops and genome instability

**DOI:** 10.1371/journal.pgen.1012040

**Published:** 2026-02-09

**Authors:** María E. Soler-Oliva, Rocío A. Domínguez-Sierra, Hélène Gaillard, Andrés Aguilera

**Affiliations:** 1 Centro Andaluz de Biología Molecular y Medicina Regenerativa - CABIMER, Consejo Superior de Investigaciones Científicas - Universidad de Sevilla - Universidad Pablo de Olavide, Seville, Spain; 2 Departamento de Genética, Facultad de Biología, Universidad de Sevilla, Seville, Spain; National Institute of Environmental Health Sciences, UNITED STATES OF AMERICA

## Abstract

Transcription is essential for cellular function, but it can also lead to genetic instability, particularly through the formation of secondary structures such as R-loops, which consist of an RNA-DNA hybrid and a displaced DNA strand. Unscheduled R-loop accumulation is a major source of DNA damage and has been associated with several human diseases, including cancer. While multiple factors involved in RNA biogenesis, export, and chromatin remodeling play a role in preventing R-loop accumulation, the function of essential proteins in R-loop metabolism remains unexplored. Here, we performed a genetic screening in *Saccharomyces cerevisiae* using over 1200 temperature-sensitive mutants to identify novel proteins involved in the prevention of R-loop-associated genomic instability. Our results reveal that the SWI/SNF-like protein Mot1 plays a key role in preventing R-loop accumulation and R-loop-associated genome instability. Its role is particularly important during S phase, where Mot1 dysfunction leads to R-loop dependent replication impairment, presumably due to transcription-replication conflicts (TRCs). Epistatic relationships between mutations in *MOT1* and the S-phase specific DNA-RNA helicase *SEN1* further support the role of Mot1 in TRCs. The study highlights the importance of transcriptional regulators in maintaining genome stability by mitigating TRCs and regulating R-loop homeostasis.

## Author summary

The maintenance of genome integrity is crucial to safeguard organism viability and avoid diseases. During the process of transcription, secondary structures known as R-loops, which consist of an RNA-DNA hybrid and a displaced DNA strand, may be formed. Such structures have been shown to interfere with other DNA-templated processes, including replication, thus resulting in genetic instability. Multiple factors implicated in the processes of RNA biogenesis, export, and chromatin remodeling have been shown to exert a function in the prevention of R-loop accumulation; however, the role of essential proteins in R-loop metabolism remained unexplored. In this study, budding yeast was used as eukaryotic model organism to perform a genetic screening with the aim of identifying essential proteins involved in the prevention of R-loop-associated genomic instability. We found that the Mot1 transcription factor plays a key role in preventing R-loop accumulation and R-loop-associated genome instability. Our results showed that its role is particularly important during S phase, where Mot1 dysfunction affects replication fork progression. We provide genetic and molecular evidence that these defects are primarily attributable to transcription-replication conflicts mediated by R-loops. Our study highlights the significance of transcriptional regulators in preserving genome stability by mitigating transcription-replication conflicts and regulating R-loop homeostasis.

## Introduction

During transcription, DNA undergoes topological changes that make it more susceptible to being damaged. Additionally, secondary structures may form, such as R-loops, which consist of a DNA-RNA hybrid and a displaced single-stranded DNA (ssDNA). Although R-loops may carry physiological functions in specific cases, unscheduled R-loop accumulation can lead to DNA breaks, either by exposing displaced ssDNA, which is more vulnerable to genotoxic agents and nucleases, or by blocking replication forks, leading to R-loop-driven transcription-replication conflicts (TRCs) [1–3]. R-loop accumulation is a major source of genetic instability and has been implicated in several human diseases, including cancer.

In recent years, an increasing number of factors have been identified that play a role in the prevention or resolution of R-loop accumulation. These factors include different RNA processing and export factors. A paradigmatic example is the THO complex, which is required for mRNP biogenesis at RNAPII-transcribed regions. Mutations in subunits of the THO complex cause defects in transcription elongation and RNA export in addition to DNA damage mediated by R-loops in yeast and human cells [4]. Other RNA processing and export factors, including nuclear pore proteins and splicing factors, have also been reported to function in preventing R-loop accumulation [5–9]. In addition, since R-loops are formed behind elongating RNAP, transcriptional defects during elongation that block RNAP may alter R-loop homeostasis, leading to DNA damage [10,11]. Besides proteins involved in RNA metabolism, torsional stress can also favour the formation of R-loops, and topoisomerase activities are required to prevent unscheduled R-loop accumulation [12–14]. Other factors contribute to R-loop homeostasis by acting as resolvases. Probably the most relevant and well-known factor with this function is RNase H (RNH), a conserved ribonuclease that specifically degrades the RNA moiety of the DNA-RNA hybrids [15]. Several helicases with DNA-RNA hybrid unwinding functions have been described, including Sen1 (the yeast ortholog of SETX), which acts specifically during S phase thereby preventing R-loop-mediated TRCs [16–19]. The chromatin environment also plays a critical role in mitigating these transcription-replication encounters, as chromatin remodelers such as SWI/SNF, ARID1A and INO80, along with histone chaperones such as FACT, have been shown to prevent R-loop-mediated TRCs [20–23].

Although recent genetic screenings in yeast have identified new factors with a function in R-loop metabolism, essential genes have not been systematically explored, leaving significant gaps in our understanding of R-loop homeostasis. With the aim of identifying essential proteins involved in the prevention or resolution of R-loop-associated genomic instability, we performed a genetic screening in the model organism *Saccharomyces cerevisiae* using an available collection of more than 1200 temperature-sensitive (ts) mutants [24]. We used the controlled expression of the human cytidine deaminase enzyme (AID) to exacerbate R-loop-mediated genomic instability in a recombination reporter construct. This approach allowed us to identify the SWI/SNF-like protein Mot1 as a novel factor involved in preventing of DNA damage associated with R-loop accumulation. Defective Mot1 function has been associated with impaired transcription elongation [25] and increased pervasive transcription [26,27] which has been proposed to interfere with replication initiation [28]. Here, we show that Mot1 dysfunction leads to R-loop accumulation and subsequent genome instability. Mot1-depleted cells accumulate R-loops mainly during S phase and show R-loop-dependent replication impairment, likely associated with TRCs. Moreover, *mot1* and *sen1* temperature-sensitive (ts) mutations are epistatic with respect to genetic instability. These findings highlight the importance of transcriptional regulators, such as Mot1, in maintaining R-loop homeostasis and in mitigating TRCs by acting during DNA replication.

## Results

### Genetic screening of essential genes that suppress R-loop-induced genome instability

**Fig 1:**
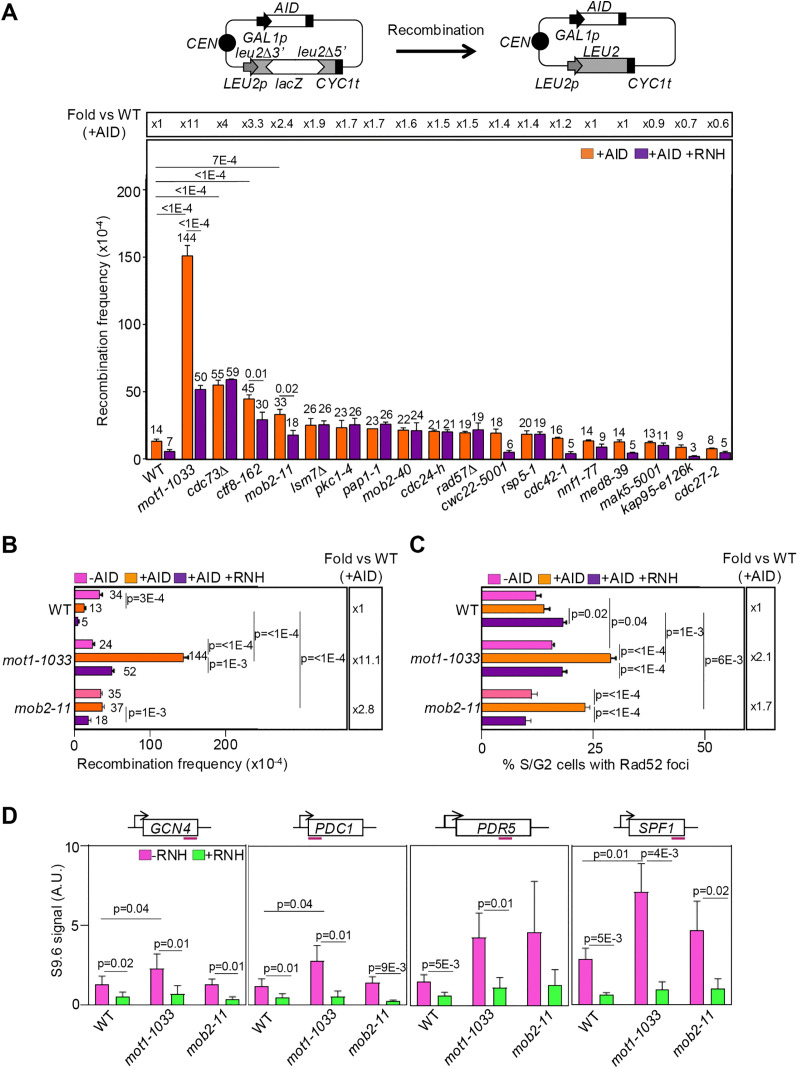
R-loop-mediated genomic instability in *mot1-1033* and *mob2-11* cells. (A) Recombination analyses in the indicated strains from the ts collection transformed with p313LZGAID. A scheme of the plasmid and GL-LacZ recombination system is shown (top). Cells were also transformed with pRS416GALRNH1 (+AID + RNH, purple) or pRS416 (+AID, orange) to overexpress or not RNase H1. Transformed cells were cultured at 32ºC in galactose to express AID, and recombination frequencies obtained as the median value of six independent colonies. Average and SEM are shown for each genotype (n = 3). Statistical analyses were performed using a two-way ANOVA followed by Holm-Šidák’s multiple comparisons test. Only significant p-values are shown. (B) GL-lacZ recombination analyses in WT, *mot1-1033* and *mob2-11* cells cultured in glucose (no AID expression, -AID, pink) or galactose (AID expression, + AID). Further details and statistical analysis as in (A). (C) Percentage of S/G2 cells with Rad52-YFP foci in cells transformed with pWJ1344 and cultured at 32ºC. Average and SEM of independent experiments in which at least 200 cells were analysed are shown (n = 3). Details as in (B), statistical analysis as in (A). (D) DNA-RNA hybrid immunoprecipitation (DRIP) using the S9.6 antibody at the *GCN4*, *PDC1*, *PDR5* and *SPF1* genes in WT, *mot1-1033* and *mob2-11* asynchronous cultures cultured at 32ºC. The location of the qPCR amplicons is indicated for each locus (top). Samples were treated (+RNH, green) or not (-RNH, pink) with RNase H *in vitro* prior to immunoprecipitation. Average and SEM of independent experiments are shown (n ≥ 5). Statistical analyses were performed using independent two-tailed paired Student’s t-tests to assess both genotype differences (Mutant vs. WT) and signal specificity (-RNH vs. + RNH), treating the + RNH condition as an internal negative control. Each genomic locus was analysed independently to account for locus-specific variations in baseline R-loop levels. Only significant p-values are shown.

Although a handful of genetic screenings have been recently carried out to identify new agents involved in R-loop homeostasis [5,29–32], essential genes have so far not been systematically explored. We took advantage of a collection of 1232 ts mutants in essential genes from *S. cerevisiae* [24] to seek for proteins with a role in the prevention of R-loop-mediated genome instability. For this, the human AID enzyme was ectopically expressed to exacerbate R-loop-mediated recombination. AID deaminates cytosine residues in the ssDNA of the R-loops, creating uracil residues that are subsequently processed by DNA repair pathways, resulting in DNA breaks [33,34]. This strategy has been successfully employed to identify suppressors of R-loop-mediated genome instability [5,30,33]. The ts mutant strain collection was transformed with a plasmid that contains the human *AID* gene driven by the *GAL1* promoter (*GAL1p*) and a recombination system consisting in two direct repeats of truncated *leu2* genes with the *LacZ* bacterial gene in the intervening sequence (GL-LacZ) (Fig 1A, top). For each strain, three transformant isolates were cultured at 27ºC and at 32ºC in solid media containing galactose to induce the expression of AID and recombination assessed by comparing growth of total and recombinant cells (S1A Fig). 250 strains showing similar number of recombinants as *mft1∆* and *mlp1∆*, which were used as positive controls, were selected for further analysis. Since all candidates were able to grow at 32°C, all subsequent steps of the genetic screening were performed at this temperature. Next, transformants from the selected strains were grown with and without AID expression and serial dilutions were plated to evaluate the contribution of AID on recombination (S2B Fig). The strains were classified in the following categories: low level of basal recombination that increased upon AID expression (Cat. I, 42 strains), high level of basal recombination that increased upon AID expression (Cat. II, 28 strains), and high level of recombination independently of AID expression (Cat. III, 50 strains) (S1 Table). Recombination analyses with and without AID expression were then performed in the GL-LacZ system for candidates from categories I and II bearing mutations in genes that encode nuclear proteins (S2 Table). This led to the selection of 18 strains showing AID-dependent hyper-recombination, which were then subjected to recombination analyses with or without concomitant RNase H1 (RNH1) over-expression to assess whether the action of AID relies on the accumulation of R-loops. A statistically significant increase in recombination as compared to the wild-type (WT) was observed in four strains, with this increase being sensitive to RNH1 expression in three of the strains: *mot1–1033, ctf8–162,* and *mob2–11* (Fig 1A). Recombination in cells carrying the *mot1–1033* allele increased over tenfold over the wild-type levels, while the increase observed in the *mob2–11* and *ctf8–162* strains was moderate. These results suggest that the *MOT1*, *CTF8,* and *MOB2* gene products may play a role in the prevention of R-loop-mediated genome instability.

### *mot1–1033* leads to genomic instability and R-loop accumulation

We further analysed the *mot1–1033* strain, which showed the strongest phenotype, and added the *mob2–11* strain as one candidate with a mild phenotype. Recombination analyses were performed at 32ºC using the GL-LacZ system with and without AID and RNH1 expression (Fig 1B). Both mutants showed a significant increase in recombination frequency as compared to WT that was dependent on AID expression and significantly reduced upon RNH1 over-expression. Next, we used Rad52-YFP foci as a marker of double strand breaks (DSBs) to assess whether these mutations cause genomic instability by fluorescence microscopy (Fig 1C). The assay was performed in cells cultured at 32ºC. As compared to WT cells, *mot1–1033* and *mob2–11* cells both displayed a ~ 2-fold increase in the percentage of S/G2 cells with Rad52-YFP foci when AID was expressed, which was significantly decreased upon RNH1 overexpression. Worthy of note, a slight yet statistically significant increase in cells with Rad52-YFP foci was observed in the absence of AID overexpression in *mot1–1033* cells. Altogether, these results support that these mutants display R-loop-mediated genome instability. Then, R-loop levels were assessed at the molecular level by DNA:RNA hybrid immunoprecipitation (DRIP) with the S9.6 antibody at the *GCN4*, *PDC1*, *PDR5* and *SPF1* genes, which were previously reported to accumulate R-loops [5]. A significant increase in S9.6 signal with respect to WT was observed at three out of the four analysed genes in *mot1–1033* cells cultured at 32ºC (Fig 1D). The specificity of the S9.6 signal was confirmed by *in vitro* treatment with recombinant RNH. On the contrary, *mob2–11* cells displayed similar S9.6 signal as WT cells at all four regions. It is likely that this mutant enhances recombination as an outcome of R-loops accumulated at WT levels. To complete this first characterization, we performed flow cytometry analyses (S1C Fig). While *mot1–1033* showed a slight enrichment in G1 cells, compatible with defects in early S phase, *mob2–11* cells exhibited an accumulation of cells in G2/M, consistent with the role of Mob2 in mother/daughter cell separation [35]. Thus, given the absence of R-loop accumulation and the altered cell cycle distribution of *mob2*–11 cells, this mutant was discarded from further analysis.

To pursue our analysis of the *mot1–1033* mutation in the W303 genetic background, we first assessed recombination of the GL-LacZ system and Rad52-YFP foci formation with and without concomitant expression of AID and RNH1 (S2A and S2B Fig). Due to the increased thermo-sensitivity of the mutant in this genetic background, all experiments were performed with cells cultured at 30ºC. Whereas Rad52 foci analysis gave similar results as in the BY4741 genetic background, *mot1–1033* cells showed an increase in recombination frequency even when AID was not expressed. AID expression did not enhance recombination further, but RNH1 overexpression led to a significant reduction of recombination frequency, indicating that the increase in recombination caused by *mot1–1033* was mediated by R-loops. Consistently, *mot1–1033* cells displayed a significant increase in S9.6 signal at the *GCN4* and *PDC1* genes as assessed by DRIP (S2C Fig). Together, our results indicate that *mot1–1033* accumulates R-loops and leads to genomic instability.

### *mot1–1033*-driven genomic instability depends on transcription and R-loops

**Fig 2:**
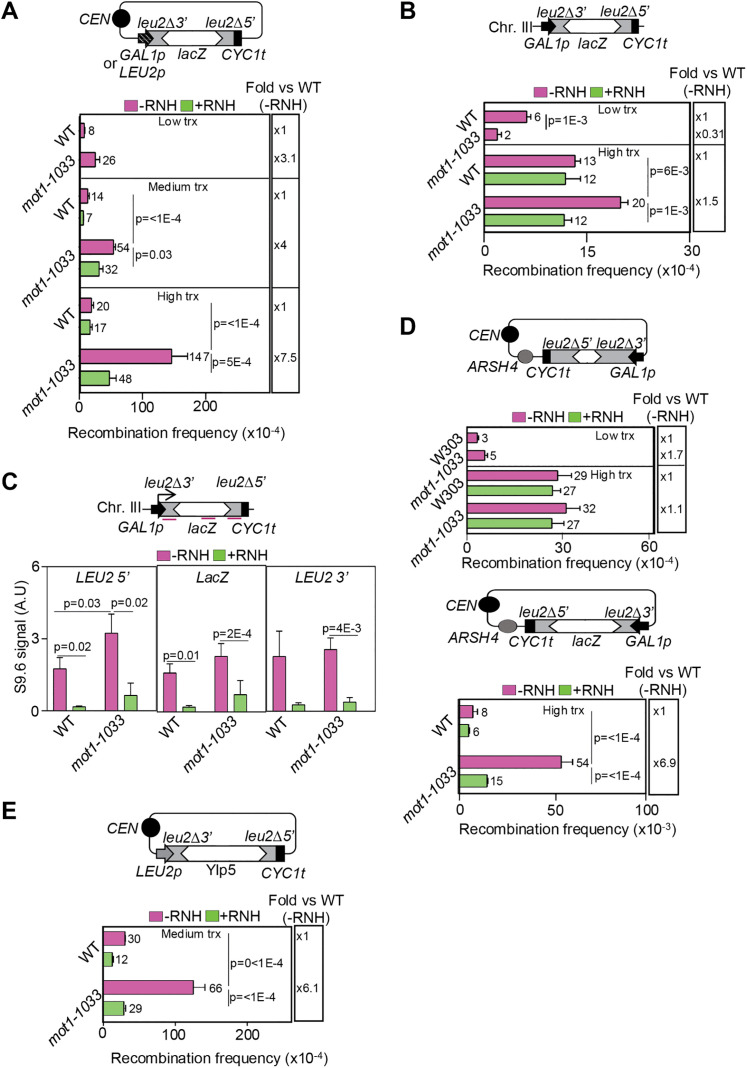
*mot1*–1033 genomic instability depends on transcription and R-loop accumulation. (A) Recombination analyses in WT (W303a) and *mot1–1033* (mot1–1033-W303-a) cells cultured at 30ºC using the plasmid-borne direct repeat L-lacZ system expressed under the control of the *GAL1* promoter in glucose or galactose (low and high transcription levels, respectively) or under the control of the *LEU2* promoter (galactose, medium transcription level). A schematic representation of the recombination system is shown (top). pRS416 and pRS416-GALRNH1 plasmids were used to express (green, + RNH) or not (pink, -RNH) the RNase H1 enzyme. Average and SEM of fluctuation assays from six independent colonies are plotted (n = 3). Statistical analyses were performed using a two-way ANOVA followed by Holm-Šidák’s multiple comparisons test, except for WT vs *mot1–1033* under low transcription conditions, which was analysed using one-way ANOVA. Only significant p-values are shown. The fold increase of each mutant relative to the WT in the -RNH condition is shown. (B) Recombination analyses of WT (WGLZN) and *mot1–1033* (WGLZN-mot1–1033) cells expressing L-lacZ from the *GAL1* promoter at the endogenous *LEU2* locus on chromosome III (GL-lacZ). Cells were cultured at 30ºC in galactose or glucose (low and high transcription levels, respectively). Further details and statistical analysis as in (A). (C) DRIP using the S9.6 antibody in WT and *mot1–1033* at the *LEU2* 5’, *LacZ* and *LEU2* 3’ positions of the GL-LacZ chromosomal recombination system. Asynchronous cell cultures were grown at 30ºC and recovered 90 min after transcription induction by galactose addition. Amplicon’s locations are highlighted (top). Samples were treated (green, + RNH) or not (pink, -RNH) with RNase H in vitro prior to immunoprecipitation. Average and SEM of independent experiments are plotted (n = 5). Statistical analyses were performed as described in the legend of Fig 1D. Only significant p-values are shown. (D) Recombination analyses using the plasmid-borne direct repeat *leu2* system expressed under the control of the *GAL1* promoter in head-on orientation with respect to the *ARSH4* sequence with (bottom) or without (top) intervening *LacZ* sequence. Other details and statistical analysis as in (A). (E) Recombination analyses using the LY recombination system expressed under the control of the *LEU2* promoter. Details and statistical analysis as in (A).

To assess whether *mot1–1033*-driven recombination depends on transcription, we used the GL-LacZ system under low and high transcription conditions (glucose and galactose, respectively). Intermediate transcription conditions were tested in the same recombination reporter system under the constitutive *LEU2* promoter (*LEU2p*, L-LacZ). Cells were cultured at 30º for these recombination assays. No difference between WT and *mot1–1033* cells was observed at low transcription levels, whereas *mot1–1033* cells showed a 4-fold increase as compared to WT at intermediate transcription levels that was further enhanced to 7.5-fold at high transcription levels (Fig 2A). Importantly, the observed increases in recombination frequency were suppressed by RNH1 overexpression. In agreement with these results, *mot1–1033* cells showed hyper-recombination only when transcription levels were high using a chromosomal GL-LacZ recombination system (Fig 2B). Again, this increased recombination was significantly reduced upon RNH1 overexpression, indicating that it was mediated by R-loops. To make sure that the *mot1–1033* mutation does not affect the expression of the different recombination reporters, we quantified the amount of *LEU2* mRNA by RT-qPCR in the recombined systems and found no significant differences between WT and *mot1–1033* cells (S3 Fig). In addition, we directly assessed R-loop levels at the GL-LacZ chromosomal system by DRIP (Fig 2C). A significant increase in DNA:RNA hybrids was observed at the 5’-end of the recombination system in *mot1–1033* cells cultured at 30ºC, consistent with the idea that the progression of RNAPII is hampered in the absence of a fully functional Mot1 but leading to DNA-RNA hybrids at the beginning of the transcriptional unit. Altogether, these results indicate that the *mot1–1033* allele leads to genomic instability that is dependent on both transcription levels and R-loops.

Since R-loop accumulation has been reported to occur preferentially in long, high GC content and highly transcribed genes [36], we investigated whether the *mot1–1033* allele also leads to increased recombination in systems that do not contain *LacZ*, a long bacterial gene (3 kb) with a high GC content (56%) and thus prone to R-loop formation. We used a recombination system containing two truncated *leu2* direct repeats separated by a short DNA sequence (31 bp) under the control of *GAL1*p and oriented in head-on (HO) orientation with respect to replication, since this is the most sensitive orientation to detect TRCs [37]. As a control, we used the same recombination system but with the *LacZ* sequence in between the *leu2* repeats [38]. Cells were cultured at 30ºC for these experiments. In the system lacking *LacZ*, the recombination frequencies obtained in *mot1–1033* and WT cells were comparable even under conditions of high transcription (Fig 2D). However, the presence of *LacZ* in between the *leu2* repeats caused a 6.9-fold increase in recombination frequency in *mot1–1033* cells above the already high WT basal level. To gain further insight into the sequence requirements for *mot1–1033*-driven hyper-recombination, we used the LY recombination system [39], in which the intervening sequence between the *leu2* repeats consists in the Ylp5 vector (5.5 kb, 50% GC). In this case, we observed an increase in recombination in *mot1–1033* as compared to WT that was again around 6-fold (Fig 2E), even under medium transcription levels, the system being under the control of the *LEU2p*. Our results suggest that Mot1 prevents genomic instability, but this effect was preferentially detected in genes with specific features that make them prone to R-loop accumulation, such as large size, high GC content or high transcription levels.

### Degron-mediated depletion of Mot1 lead to R-loop-dependent genomic instability

**Fig 3:**
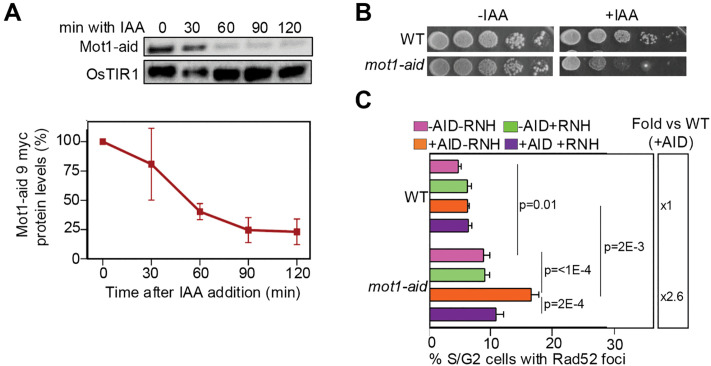
Genome instability in a newly constructed auxin-inducible Mot1 degron strain. (A) Representative Western blot (top) showing Mot1 depletion at different timepoints after the addition of 1 mM auxin (IAA) in *mot1-aid* cultured at 30ºC. Anti-myc antibody was used to detect Mot1 aid-9myc and OsTIR-9myc, which was used as loading control. Percentage of protein levels was measured relative to timepoint 0 (bottom). Average and SEM of independent experiments are plotted (n = 3). (B) Growth assay of WT and *mot1-aid* cells. 10-fold serial dilutions of exponentially growing cultures were plated on YPAD supplemented with 1 mM auxin (right, + IAA) or not (left, -IAA) and incubated for 2 days at 26ºC to minimize auxin degradation. (C) Percentage of S/G2 cells containing Rad52-YFP foci in WT and *mot1-aid* cells cultured at 30ºC and treated with 1 mM auxin (NAA) for 2 h. pRS317-GALRNH1 and pRS317 plasmids were used to express or not RNase H1 (RNH1). pRS413-GALAID and pRS413 plasmids were used to express or not the AID enzyme. Cells were transformed with different combinations of plasmids to obtain four experimental conditions: without AID and RNH1 expression (-AID -RNH, pink), with RNH1 expression only (-AID + RNH, green), with AID expression only (+AID -RNH, orange), and with both AID and RNH1 expression (+AID + RNH, purple). Average and SEM of independent experiments in which at least 200 cells were analysed are plotted (n = 4). Statistical analyses were performed using a two-way ANOVA followed by Holm-Šidák’s multiple comparisons test. Only significant p-values are shown. Fold increase relative to WT in +AID -RNH conditions is shown.

As Mot1 is an essential protein with ubiquitous roles in transcription, we constructed a Mot1 auxin-inducible degron (aid) strain (*mot1-aid*) to appraise the immediate consequences of Mot1 depletion as well as to confirm that the R-loop-mediated genome instability was not allele-specific. Mot1 was efficiently depleted upon auxin treatment, although residual Mot1 could still be detected by Western blot at later time-points (Fig 3A). Quantification indicated that about 23% of the initial signal persisted after two hours of auxin treatment. To achieve near-complete depletion, it was necessary to extend the treatment to four hours, reducing residual Mot1 levels to 4% (S4A Fig). Consistent with the essential function of Mot1, growth assays in solid media supplemented or not with 1 mM auxin showed a severe growth impairment of *mot1-aid* cells only upon auxin exposure (Fig 3B). We then assessed whether Mot1 depletion causes an increase in DSBs by measuring the percentage of cells with Rad52-YFP foci in auxin-treated cell cultures with and without concomitant AID and RNH1 expression (Fig 3C). In the absence of AID expression, Mot1-depleted cells cultured at 30ºC displayed a higher percentage of cells with repair foci after two hours of auxin treatment as compared to the WT (1.8-fold). This increase was not suppressed by RNH1 overexpression. However, when AID was expressed, the fold change increased to 2.6, and this AID-dependent increase was suppressed by RNH1 overexpression. Extending auxin treatment to four hours caused a 10-fold increase in the percentage of cells with Rad52 foci compared to WT, even in the absence of AID expression, and this increase was sensitive to RNH1 overexpression (S4B Fig). Similarly, DRIP analysis after four hours of auxin treatment revealed a significant increase in R-loop levels at the *GCN4* and *PDC1* loci, with a trend toward increased signal at *SPF1* that did not reach statistical significance (S4C Fig). Expression of these loci was assessed by RT-qPCR and indicated that Mot1 depletion does not alter their transcription (S4D Fig). These results indicate that Mot1 depletion per se leads to R-loop-mediated genome instability.

### Accumulation of R-loops upon Mot1 depletion occurs preferentially in S phase

Several factors that prevent R-loop accumulation have been described to function preferentially during S phase, such as SWI/SNF and Sen1/SETX [19,20]. We therefore wondered whether the role of Mot1 in preventing R-loops might be restricted to S phase. To address this question, DRIP was performed in asynchronous, G1 synchronised and S phase-enriched auxin-treated cultures at 30ºC (S5A-C Fig). These experiments were performed after two hours of auxin exposure, as cell cycle phase enrichment cannot be achieved with longer treatment. However, no significant differences between WT and *mot1-aid* cells were observed under these conditions.

**Fig 4:**
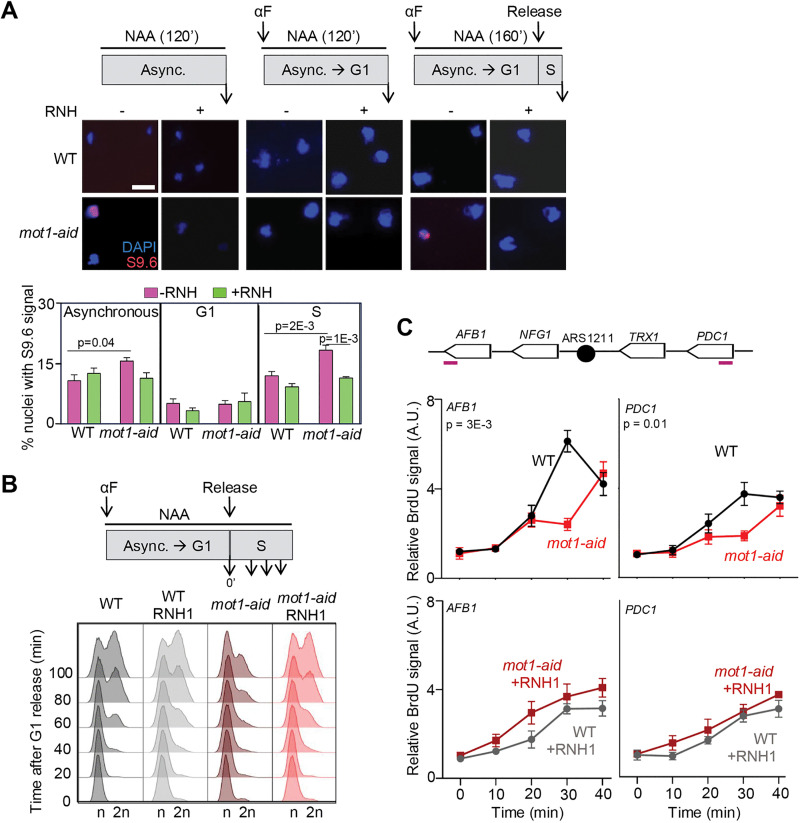
Mot1 depletion leads to R-loop accumulation during S phase and to R-loop-dependent DNA replication impairment. (A) S9.6 immunofluorescence (red) of chromosome spreads from asynchronous, G1-synchronised and S-phase enriched WT and *mot1-aid* cultures grown at 30ºC and treated with 1 mM auxin (NAA) for 2 h. A scheme of the experimental design (top) and representative images are shown. Cells were transformed with pRS413-GALRNH1 or pRS413 and cultured in galactose-containing medium to induce RNase H1 expression (+) or not (-). DNA was stained with DAPI (blue). Scale bar = 5 μm. Quantification of the percentage of nuclei with S9.6 signal is shown as average and SEM of independent experiments in which at least 100 nuclei were analysed (n = 3) (bottom). Statistical analyses were performed using a two-way ANOVA followed by Holm-Šidák’s multiple comparisons test. Only significant p-values are shown. (B) Flow cytometry analyses of WT and *mot1-aid* cells during release from G1 arrest. Schematic representation of the experiment is depicted (top). WT and *mot1-aid* cell cultures were treated with 1 mM NAA for 2 h during α-factor G1 synchronisation and then released into fresh media supplemented with 1 mM NAA. Other details as in (A). (C) Analysis of replication by BrdU ChIP in WT and *mot1-aid* strains at two regions proximal to ARS1211 where transcription and replication occur in co-directional (*AFB1*) or head-on (*PDC1*) orientation. Experiments were performed as in (B) with BrdU added at timepoint 0’. A schematic representation of regions analysed, and the location of the amplicons is shown (top). Relative enrichment as compared to a late-replicating region of chromosome V were calculated using the 2^-∆∆Ct^ method. Average and SEM of independent experiments are shown (n = 3 or 4). Statistical analyses were performed using a two-way ANOVA considering the interval between timepoints 10 and 30 min. Only significant p-values are shown.

To evaluate global R-loop accumulation throughout the cell cycle, we then performed S9.6 immunofluorescence in chromosome spreads, which allows detection of global changes in DNA-RNA hybrid accumulation. Asynchronous, G1 synchronised, and S phase-enriched wild-type and *mot1-aid* cultures were analysed after two hours of auxin treatment (Fig 4A). In asynchronous *mot1-aid* cultures, a slight increase in cells with S9.6 signal was observed compared to WT, which was partially suppressed by RNH1 overexpression. Whereas G1 synchronised cultures of *mot1-aid* cells did not display any increase with respect to WT, Mot1 depletion caused a significant increase in the number of cells with S9.6 signal with respect to WT in S phase-enriched cultures. RNH1 overexpression completely suppressed the increase in S9.6 signal, indicating that the signal detected was specific for DNA-RNA hybrids. Western blot analysis confirmed that G1 synchronization did not alter the extent of Mot1 depletion achieved upon auxin treatment (S6A Fig). These results indicate that Mot1 depletion leads to R-loop accumulation preferentially in S phase.

### Degron-mediated Mot1 depletion leads to replication impairment

Since R-loop accumulation is known to interfere with replication and to be a cause of genome instability [1], we wondered whether Mot1 depletion, which causes accumulation of R-loops mainly in S phase, might affect replication. We first used flow cytometry analysis to follow cell cycle progression upon release from G1 arrest in cells depleted or not of Mot1 (Fig 4B). Auxin treatment was initiated two hours before release from α-factor arrest and maintained throughout the experiment. Mot1-depleted cells exhibited a delay in S-phase entry as compared to the WT, which was suppressed by RNH1 overexpression. These results suggest that Mot1 depletion affects cell cycle progression in an R-loop-dependent manner. To analyse replication at the molecular level, incorporation of the thymidine analogue BrdU was followed by ChIP-qPCR at regions where replication encounters transcription in a co-directional (*AFB1*) or HO (*PDC1*) orientation near the early replication origin ARS1211. The genes located at these regions are transcribed in G1 cells, albeit with different expression levels (S6B Fig). BrdU incorporation at both regions was delayed in *mot1-aid* cells as compared to WT (Fig 4C). A delay was also observed at the ARS1211 locus in *mot1-aid* cells, suggesting a general impairment of replication in the absence of Mot1 (S6C Fig). Overexpression of RNH1, which is predicted to act on Okazaki fragments in addition to its activity on R-loops, led to a decrease in BrdU incorporation in WT cells, as previously documented [5]. In replication-impaired *mot1-aid* cells, BrdU incorporation was slightly increased by RNH1 overexpression, suggesting that R-loop removal improves the replication capacity in the absence of Mot1. The difference in BrdU incorporation observed between WT and *mot1-aid* was lost at the three regions in cells overexpressing RNH1, supporting that R-loop accumulation contributes to the replication delay. Collectively, these results indicate that Mot1 depletion gives rise to replication impairment mediated by R-loop accumulation, supporting a role for Mot1 in the prevention of R-loop-dependent TRC.

### Mot1 dysfunction is epistatic with S phase-specific Sen1 mutations

**Fig 5:**
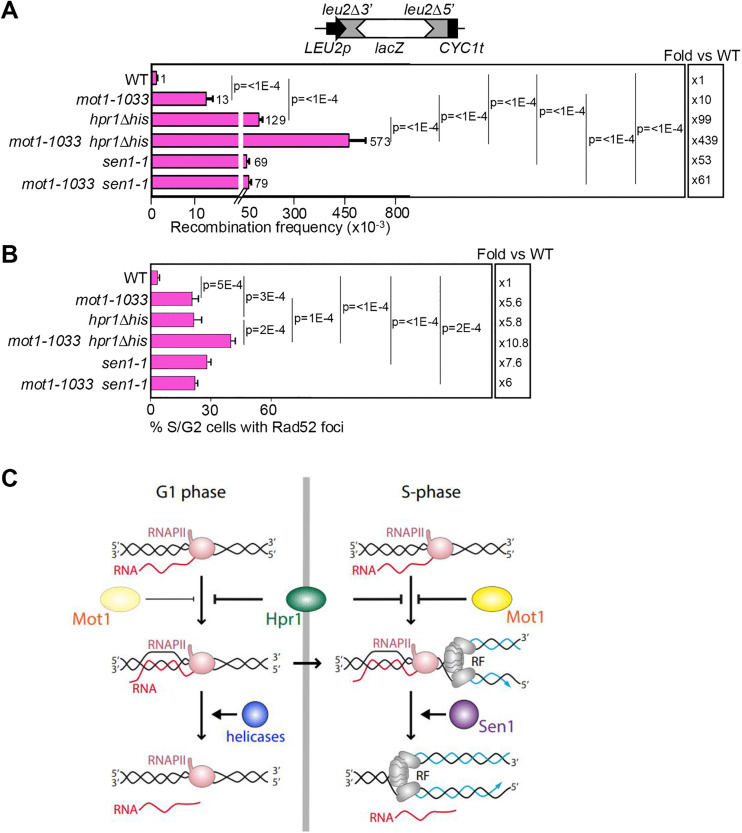
Genetic interactions between *mot1-1033*, *hpr1Δ* and *sen1-1* mutants. (A) Recombination analyses using the L-LacZ recombination system in WT, *mot1-1033*, *hpr1Δ*, *mot1-1033 hpr1Δ*, *sen1-1* and *mot1-1033 sen1-1* cells cultured at 30ºC. Average and SEM of fluctuation tests from six independent colonies are shown (n = 3). Statistical analyses were performed using a one-way ANOVA on log-transformed data, followed by Holm-Šidák’s multiple comparisons test. Only significant p-values are shown. The fold increase relative to WT is shown for each mutant (right). (B) Percentage of S/G2 cells containing Rad52-YFP foci in the indicated strains. Average and SEM of independent experiments in which at least 200 cells were analysed are plotted (n = 3). Statistical analyses were performed using a one-way ANOVA followed by Holm-Šidák’s multiple comparisons test. Details as in (A). (C) Model for Mot1 function in preventing R-loop accumulation during S phase. Whereas the Hpr1 subunit of the THO complex is a general safeguard against R-loop accumulation, we propose that Mot1 prevents the accumulation of R-loops mainly in S phase, likely acting upstream of the Sen1 resolvase. Modified from an original drawing published in [19] under a CC BY 4.0 license http://creativecommons.org/licenses/by/4.0/.

Several transcription-associated factors are involved in the prevention of deleterious R-loop accumulation, but their role may not be the same in all phases of the cell cycle. Among them, the THO complex subunit Hpr1 acts as a general safeguard against R-loop formation mainly during G1, whereas the Sen1 helicase acts specifically by unwinding DNA-RNA hybrids formed during S phase [16,19]. To genetically address whether *mot1* interacts with mutations in these G1- or S-phase specific factors, and as an alternative approach to confirm whether Mot1 preferentially protects against R-loops in S phase, we generated *mot1–1033 hpr1Δ* and *mot1–1033 sen1–1* double mutants and performed recombination analyses using the L-LacZ recombination system in cells cultured at 30ºC (Fig 5A). Interestingly, the *mot1–1033 hpr1Δ* double mutant showed a recombination frequency more than 40-fold higher than *mot1–1033* cells and more than 4-fold higher than *hpr1Δ* cells. In contrast, *mot1–1033 sen1–1* double mutants showed a similar level of recombination to *sen1–1* single mutants. Next, the percentage of cells with Rad52-YFP foci was measured after a heat-shock of 3h at 37ºC in *mot1–1033, hpr1*Δ, *sen1–1* and the corresponding double mutants (Fig 5B). Notably, these experimental conditions significantly increased the percentage of Rad52-YFP foci in *mot1–1033* cells compared to the WT without the need for AID expression. In line with the results of the recombination analyses, *mot1–1033 hpr1Δ* cells showed a greater accumulation of foci than the single mutants, whereas *mot1–1033 sen1–1* displayed a similar percentage of Rad52 foci with respect to the *mot1–1033* strain. Altogether, these results point out to a synergistic/additive effect of *mot1–1033 and hpr1Δ* mutations, whereas the outcome of *mot1–1033 and sen1–1* mutations with respect to transcription-dependent genomic instability is epistatic, consistent with a function of Mot1 to protect against R-loop accumulation and R-loop-mediated genome instability during S phase.

## Discussion

Unscheduled accumulation of R-loops has been linked with several diseases including neurodegenerative disorders and cancer [36]. Although several factors that contribute to the prevention or resolution of R-loops have been described over the past decade [5–8,20,22,23,29], our current understanding of the mechanisms that protect cells from harmful R-loops remains limited. In this study, we identified the essential SWI/SNF-like ATPase Mot1 as a novel factor with a role in preventing R-loop-mediated genetic instability. We show that R-loop accumulation occurs specifically in S phase in Mot1-depleted cells, which also exhibit replication fork progression impairment. The function of Mot1 in preventing R-loops in replicating cells is further supported by the epistatic genetic interaction of its mutation with that in the Sen1 helicase, which has been shown to remove R-loops during S phase [16,19], and the synergistic relation with the *hpr1* mutation of the THO complex, which prevents R-loops preferentially in G1. Based on these results, we propose a model in which Mot1 prevents the accumulation of R-loops mainly during S phase, thereby contributing to the avoidance of deleterious TRCs and R-loop-mediated genetic instability (Fig 5C).

The transcription factor Mot1 can act both positively and negatively on gene expression through its ability to dissociate DNA-bound TBP by ATP hydrolysis [40]. However, the efficiency of this reaction has been shown to be reduced, often requiring multiple catalytic events or Mot1 molecules to displace a single TBP *in vitro* [41]. Further evidence indicates that the function of Mot1 is not limited to TBP displacement, including nucleosome remodelling at both promoter and non-promoter sites in cooperation with the SAGA complex subunit Spt3 [42] and the appropriate positioning of +1 and +2 nucleosomes at the 5’ ends of genes independent of whether their expression is regulated by Mot1 [43]. While the expression of the reporter systems used in our study was not altered by Mot1 depletion, DNA-RNA hybrids were detected at the 5′ region of the chromosomal GL-LacZ recombination system, suggesting that Mot1 may be required for efficient transcription of the GC-rich *LacZ* sequence. Interestingly, R-loop formation has previously been reported in mutants with elongation and/or mRNP biogenesis defects including the THO complex and the TFIIS elongation factor [10,44]. Previous work has shown that Mot1 prevents premature termination of sense transcripts [25,43] and represses pervasive transcription in coordination with the NC2 transcription and the INO80 remodelling factors [27,45]. Thus, defects in these Mot1 functions may hamper RNAPII progression and favour R-loop formation.

Regulation of transcriptional activity in replicating cells is crucial to minimize the deleterious impact of TRCs and avoid genomic instability [46], as exemplified by mutations in the RNAPII subunit Rpb1, which have been shown to provoke transcription-dependent replication impairment [11]. Importantly, our results show that Mot1-depleted cells accumulate R-loops during S phase and exhibit replication fork progression impairment, suggesting that Mot1 is required to prevent transcriptional stress leading to TRCs. Recent studies have shown that Mot1 and INO80 act together to prevent pervasive transcription and that this function is required to avoid DNA breaks presumably associated to TRCs at replication origins [28]. Our study expands this model by demonstrating that the role of Mot1 in preventing genome instability is not restricted to ARS regions but also extends to protein-coding genes. Notably, we show that the DNA damage observed within genes depends on R-loop accumulation and that the lack of Mot1 is sufficient to generate R-loop-dependent replication impairment. Whether or not the underlying mechanism of R-loop accumulation is related to increased cryptic transcription remains to be investigated. Mot1 depletion did not alter the expression levels of R-loop accumulating genes, but cryptic transcription in these regions may be enhanced, contributing to R-loop accumulation, DNA damage, and replication impairment. The observation that *mot1–1033* and *sen1–1* show an epistatic interaction may support this idea, as Sen1 also acts to limit pervasive transcription through its role in the Nrd1–Nab3–Sen1 complex, which facilitates early termination of non-coding transcription [47]. Noteworthy, the interaction between Mot1 and Sen1 could also be explained by the role of Sen1 as an S-phase specific resolvase, which plays a major role in the removal of DNA:RNA hybrids at TRCs [19,48]. Indeed, both Mot1 and Sen1 physically interact with the replication-associated protein Ctf4, which has been shown to be important for the recruitment of Sen1 to the replisome [17,49]. Given that Mot1 physically interacts with several chromatin-associated factors with a role in TRCs including FACT, RSC and INO80 [43,49], and given its described role in nucleosome remodeling beyond TBP displacement [42], it is conceivable that the involvement of Mot1 in the prevention of R-loop-dependent genome instability extends beyond the prevention of pervasive transcription. Instead, Mot1 may also use ATP hydrolysis to displace DNA-bound factors other than TBP, thereby contributing to the establishment of a chromatin environment that supports replication and protects against TRC-mediated genome instability. The importance of chromatin state for genome stability is highlighted by recent work showing that the SWI/SNF subunits BRG1 and ARID1A, key components of the BAF complex, prevent R-loop accumulation during S phase in human cells, particularly at TRC sites [20,21]. In this context, it will be interesting to determine whether BTAF1, the human homolog of Mot1, shares this function, especially considering that the role of other factors such as Sen1 and FACT in R-loop-mediated TRCs is evolutionarily conserved [48,50]. In support of this idea, loss of BTAF1 has been associated with increased sensitivity to replication stress-inducing agents such as cisplatin and the DNA polymerase α inhibitor CD437 [51,52], suggesting a potential conservation of Mot1 function in TRC prevention.

Our findings reveal a novel role for Mot1 in safeguarding genome stability during replication by preventing the accumulation of deleterious R-loops. This study not only advances our understanding of the molecular mechanisms underlying R-loop homeostasis but also establishes a new functional dimension for Mot1, linking its transcriptional role to replication-associated genome stability.

## Materials and methods

### Yeast strains, plasmids and growth conditions

The yeast strains used for the genetic screening are listed in S3 Table. The genotype is BY4741 (*MAT*a *his3*Δ*1 leu2*Δ*0 met15*Δ*0 ura3*Δ*0*) and each allele is linked to a *KAN*MX resistance cassette. All other strains used in this work are listed in S4 Table. The strain mot1–1033-W303-a was obtained by three consecutive crosses with Ybp250. Double mutants and strains with the chromosomal GL-LacZ recombination reporter were obtained by genetic crosses. *mot1-aid* and BrdUinc strains were obtained by standard PCR-based gene replacement in the YNK54 and degron strains, respectively. Primers used for integration are listed in S5 Table. Yeast cells were grown in rich medium (YPAD; 1% yeast extract, 2% bacto-peptone, 2% glucose or galactose, 20 mg/L adenine) and transformants were grown in synthetic defined medium (SD; 0.17% yeast nitrogen base, 0.5% ammonium sulphate, 2% glucose or galactose, 0,2% drop-out mix). The plasmids used in this study are listed in S6 Table. Cells were grown at 30ºC or 32ºC as semi-restrictive temperature (W303 and BY4741 genetic background, respectively) and at 23ºC as permissive temperature.

### Yeast transformation

Yeast transformation was performed as previously described using the lithium acetate method with modifications to optimise conditions for ts mutants [53]. Cultures were grown at 23ºC in YPAD to mid-log phase. Cells were harvested by centrifugation, incubated with 100 mM LiAc for 15 min at room temperature, and incubated in transformation mix (33.5% (w/v) PEG_3350_, 100 mM LiAc, 0.5 mg/ml ssDNA, 0.003 mg/ml plasmid) overnight at 23ºC. Heat shock was applied at 37ºC for 10 min in a water bath and cells were plated on SD plates with appropriate amino acids. Plates were incubated at 23ºC for 5 days. For large scale transformation, cells were grown in 96 deep well plates covered with a breathable membrane.

### Genetic screening for AID-dependent recombination

In a first screening step, three isolates from different regions of the transformant biomass were grown on galactose-containing solid media at both 27ºC and 32ºC. Growth of recombinant and total cells was assessed by spotting serial dilutions on appropriate selective plates. Strains were considered for the second screening step if at least 2 out of the 3 replicates showed a comparable level of recombinants as the positive controls by visual inspection. Results obtained at 27ºC were only considered for strains that were not viable at 32ºC. In the second screening step, 4 isolates from the transformant biomass were grown at 32ºC on solid media containing either galactose or glucose to induce AID expression or not. Growth of recombinant and total cells was assessed by spotting dilutions on appropriate selective plates. Strains showing either a high level of basal recombination, which increased upon AID expression, or a high level of recombination independent of AID expression were selected for further analysis. Recombination frequencies were then quantified in the selected strains by fluctuation tests of 9 independent colonies derived from 3 independent transformation events. The median recombination frequency from these 9 colonies was used as a representative value. As a first criterion, mutants with a recombination frequency at least 4-fold higher than the WT under AID-expressing conditions were selected. To confirm AID dependence, strains with a fold change ratio (+AID)/(−AID) less than 4 were excluded from further analysis.

### Recombination analyses

Recombination frequencies were determined as the average value of the median frequencies obtained from at least 3 independent fluctuation tests with the indicated recombination systems in cells grown at the indicated temperature. Each fluctuation test was performed from 6 independent colonies according to standard procedures [54]. Total and recombinant cells were grown at 23ºC.

### Detection of Rad52-YFP foci

Rad52-YFP foci were visualized in cells transformed with plasmid pWJ1344 using a DM600B microscope (Leica) as previously described [55] with minor modifications. Mid-log cultures were grown at the indicated temperature. For Rad52-YFP foci detection in Fig 5B, mid-log cultures grown at 30ºC were heat-shocked at 37ºC for 3h. Cells were collected, fixed with 2.5% formaldehyde, washed three times with 100 mM K_i_PO_4_, incubated in cold 80% ethanol and resuspended in H_2_O with DAPI. At least 200 S/G2 cells were analysed for each transformant. Average values obtained from at least 3 independent transformants are plotted for each genotype.

### DNA-RNA hybrids immunoprecipitation (DRIP)

DNA-RNA Immunoprecipitation was performed as previously described [56]. Briefly, DNA was extracted from spheroplasts with chloroform:isoamylalcohol (24:1) followed by isopropanol precipitation. Precipitated DNA was spooled on a glass rod, washed twice with 70% ethanol, gently resuspended in TE and enzymatically digested with RNase III, *Hind*III, *EcoR*I, *BsrG*I, *Xba*I and *Ssp*I in an appropriate commercial buffer. After checking the efficiency of digestion of DNA, samples were split and treated with *E. coli* RNH or mock treated. 1/5 of samples volume was saved as input, and the rest was used for DNA-RNA hybrids immunoprecipitation with Protein A-Dynabeads coated with the S9.6 antibody. Real-time quantitative PCR was performed using iTaq universal SYBR Green (Biorad) with a QuantStudio 5 qPCR System (Applied Biosystems) at the indicated regions with 2 μL of immunoprecipitated DNA (IP) and 2 μL of a 50-fold dilution from input per well. Both the immunoprecipitated and input signals were quantified based on the reference standard curve. The S9.6 signal was determined by normalizing the immunoprecipitated signal to the input for each sample. The primer sequences are listed in S7 Table.

### S9.6 Chromosome spreads immunofluorescence

Immunofluorescent analyses of chromosome spreads were performed as described [57] with minor modifications. Spheroplasts were washed twice in 0.1 M MES, 1 M sorbitol, 1 mM EDTA, 0.5 mM MgCl_2_, pH 6.4. 20 μL of washed spheroplasts were mixed with 40 μL fixative solution (4% paraformaldehyde, 3.4% sucrose) on a microscope slide and then mixed with 80 μL of 1% of Lipsol for 1 min. Lysis was stopped by adding 80 μL fixative solution. Spreading was carried out using a pasteur pipet and the slides dried overnight in an extraction hood. Dried slides were washed for 30 min with 1X PBS in coplin jars and then blocked for 30 min with blocking buffer (5% BSA, 0.2% milk in 1X PBS) in a humidified chamber. Slides were incubated for 1 h with the S9.6 primary antibody (1 mg/mL in blocking buffer), washed for 30 min with 1X PBS, and incubated for 1 h in the dark with the Alexa fluor 594 secondary antibody (A21201, Invitrogen) diluted 1:1000 in blocking buffer. Primary and secondary antibody incubations were done at 23ºC in a humidified chamber. Finally, the slides were mounted with 50 μL of Vectashield (Vector laboratories, CA) with DAPI and sealed with nail polish. More than 100 nuclei were counted for each sample.

### Cell cycle synchronization and flow cytometry analysis

Cells were grown to mid-log phase and exposed to 0.125 μg/mL α-factor for 2 h. Cells were released from G1-arrest in fresh medium supplemented with 100 μg/mL pronase and samples taken at the indicated times. For flow cytometry analysis, cells were processed as previously described [58]. Briefly, 1 mL of the culture was centrifuged and resuspended in 1 mL 70% ethanol and stored at 4ºC. Cells were then washed in 50 mM sodium citrate pH 7.5, resuspended in 50 mM sodium citrate pH 7.5 and incubated at 50ºC for 1 h with 250 µg/mL RNase A and then for 1 h with 1 mg/mL proteinase K. Cells were diluted with an equal volume of 50 mM sodium citrate pH 7.5 containing 48 µg/mL propidium iodide and incubated in the dark for 30 min. Cells were briefly sonicated and analysed in a FACScalibur (Becton Dickinson, CA). For each histogram 50’000 yeast cells were analysed using the FlowJo 10.9.0 software (Becton Dickinson & Company).

### Replication analyses

Incorporation of BrdU was assessed by ChIP as described [59] with minor modifications. Cells were grown in SD-GAL-His medium and synchronized with α-factor in the presence of 1 mM NAA (Sigma) for 2 h, washed twice in prewarmed SD medium and twice in prewarmed SD-GAL-His, and released from G1 arrest in the presence of 200 μg/mL BrdU (Sigma) and 1 mM NAA (Sigma) by addition of 100 μg/mL pronase. Cell culture samples were treated with 0.1% Sodium Azide. Next, cells were lysed in an multibead shocker at 4ºC in lysis buffer (50 mM HEPES/KOH, 140 mM NaCl, 1 mM EDTA pH 8, 1% Triton X-100, 0.1% sodium deoxycholate). Chromatin was sonicated to an average size of 400–500 bp using a Bioruptor Pico (Diagenode). Samples were centrifuged to eliminate cell debris. An aliquot was processed as control of total DNA (Input) and the chromatin immunoprecipitated with anti-BrdU antibody (MBL) coated to Protein A Dynabeads (Invitrogen). Real-time quantitative PCR was performed using iTaq universal SYBR Green (Biorad) with a 7500 Real-Time PCR machine (Applied Biosystems). All PCR reactions were performed in triplicate. Relative BrdU incorporation was calculated using the formula 2^-ΔΔCt^ = 2^-((Ct INPUT target - Ct IP target) - (Ct INPUT control - Ct IP control))^ for each sample. A late replicating region of chromosome V (position 242210–242280, [60]) was used as control. The primer sequences are listed in S7 Table.

### Reverse Transcription quantitative PCR (RT-qPCR) analysis

Cells were grown as indicated and total RNA purified with RNAeasy kit (Qiagen) according to manufacturer instructions. RNA samples were treated with DNase I (Sigma) and cDNA synthesized using the SuperScript III First-Strand Synthesis System (Invitrogen). Half of the samples volume was saved prior to retrotranscription and used as negative control. Real-time quantitative PCR was performed using iTaq universal SYBR Green (Biorad) with a 7500 Real-Time PCR machine (Applied Biosystems). *SCR1* mRNA was used as internal control for comparative Ct calculation. Standard curves for all pairs of primers were performed for each analysis. All PCR reactions were performed in triplicate. The primer sequences are listed in S7 Table.

### Statistical analyses

Statistical tests were performed using GraphPad Prism software. For experiments involving two factors (genotype and RNH1 over-expression for example), data were analysed by two-way ANOVA. Multiple comparisons were controlled with Holm-Šidák’s posthoc. When only one factor was present, data were analysed by one-way ANOVA with Holm-Šidák’s correction for multiple testing when applicable. DRIP experiments were analysed with two independent paired t-tests. The − RNH versus +RNH comparison was used as an internal specificity control, and genotype effects were assessed by pairing mutants with WT processed side by side within each experiment to account for the high between-experiment variability in percentage of immunoprecipitation. Significant p-values (<0.05) are indicated in the corresponding graphs. Details regarding the number of replicates (n) and statistical methods are provided in the Fig legends.

## Supporting information

S1 Fig Genetic screening for ts mutants showing R-loop-dependent genomic instability. (A) Representative images of recombinant and total cells obtained from transformants of the ts collection during the first screening step. For each strain, three isolates from different areas of the transformant biomass were cultured in galactose at 27ºC and at 32ºC, as represented schematically (top). The position of WT (blue), positive controls *mft1Δ* (orange) and *mlp1Δ* (red), and an example of a positive candidate (green) are highlighted. (B) Representative images of recombinant and total cells obtained from ts collection transformants expressing AID (+AID) or not (-AID) during the second screening step. For each strain, four isolates from different areas of the transformant biomass were streaked in medium containing glucose (-AID) or galactose (+AID) and grown at 32ºC. Serial dilutions were plated on selective media to assess recombinant and total cells. Strains were classified into three categories: low level of basal recombination that increased upon AID expression (Cat. I, 42 strains), high level of basal recombination that increased upon AID expression (Cat. II, 28 strains), and high level of recombination independent of AID expression (Cat. III, 50 strains). WT and representative examples for categories I, II and III are shown (*tif6-ts4*, *hrr25–5001* and *cdc26–1*, respectively). The list of candidates and their assigned categories is provided in S1 Table. (C) Flow cytometry analyses of WT, *mot1–1033* and *mob2–11* cells cultured at 32ºC.
(PDF)

S2 Fig R-loop-mediated genomic instability in *mot1–1033* cells. (A) Recombination analyses in WT (W303a) and *mot1–1033* (mot1–1033-W303-a) cells transformed with p313LZGAID. Cells were cultured at 30ºC in glucose (no AID expression, -AID, pink) or galactose (AID expression, + AID). pRS416-GALRNH1 and pRS416 plasmids were used to overexpress RNase H1 (+RNH, purple) or not (orange). Average and SEM of fluctuation tests from six independent colonies are plotted (n = 3). Statistical analyses were performed using a two-way ANOVA followed by Holm-Šidák’s multiple comparisons test. Only significant p-values are shown. The fold increase relative to WT (+AID condition) is shown. (B) Percentage of S/G2 cells containing Rad52-YFP foci in WT and *mot1–1033* cells transformed with pWJ1344 and cultured at 30ºC. Average and SEM of independent experiments in which at least 200 cells were analysed are plotted (n = 3). Details and statistical analysis as in (A). (C) DRIP using the S9.6 antibody at the *GCN4*, *PDC1*, *PDR5* and *SPF1* genes in WT and *mot1–1033* asynchronous cultures cultured at 30ºC. Amplicon positions are shown (top). Samples were treated *in vitro* with RNase H (green, + RNH) or not (pink, -RNH) prior to immunoprecipitation. Average and SEM of independent experiments are shown (n = 5). Statistical analyses were performed as described in the legend of Fig 1D. Only significant p-values are shown.
(PDF)

S3 Fig Expression levels of *LEU2* reporters in *mot1–1033* cells. RT-qPCR measurement at the 3’ and 5’ ends of the *LEU2* mRNA in WT (W303a) and *mot1–1033* (mot1–1033-W303-a) strains cultured at 30ºC using recombined (A) GL-LacZ (B) L-LacZ (C) chromosomal GL-LacZ reporter systems. Values were normalized to *SCR1* mRNA levels. Schematic representation of recombined reporters and amplicons are shown above each graph. Average and SEM of independent experiments are plotted (n = 3). Statistical analyses were performed using a one-way ANOVA and no statistically significant differences were retrieved.
(PDF)

S4 Fig Genomic instability and R-loop accumulation in Mot1-depleted cells. (A) Analysis of Mot1 depletion in *mot1-aid* cells upon auxin (IAA) treatment for 4 h. Representative Western blot analysis showing Mot1 depletion after incubation with 1 mM IAA (top). Anti-myc antibody was used to detect Mot1 aid-9myc and OsTIR1–9myc, which was used as loading control. Quantification of Mot1 protein levels in cells treated with auxin (+ IAA, light red) or not (- IAA, dark red) (bottom). Average and SEM of independent experiments are plotted (n = 3). Statistical analysis was performed using a two-tailed paired Student t-test, p-value is shown. (B) Percentage of S/G2 cells containing Rad52-YFP foci in WT and *mot1-aid* strains after 4 h of treatment with 1 mM auxin. pRS413-GALRNH and pRS413 plasmids were used to express RNase H1 (+RNH, green) or not (-RNH, pink). Average and SEM of independent experiments in which at least 200 cells were analysed are plotted (n = 4). Statistical analyses were performed using a two-way ANOVA followed by Holm-Šidák’s multiple comparisons test. Only significant p-values are shown. (C) DRIP using the S9.6 antibody in asynchronous WT and *mot1-aid* cultures treated with 1 mM auxin for 4 h. The signal obtained at the *GCN4*, *PDC1* and *SPF1* genes is plotted. Amplicon positions are shown (top). Samples were treated *in vitro* with RNase H (green, + RNH) or not (pink, -RNH) prior to immunoprecipitation. Average and SEM of independent experiments are shown (n = 5). Statistical analyses were performed as described in the legend of Fig 1D. Only significant p-values are shown. (D) RT-qPCR measurement of *GCN4*, *PDC1* and *SPF1* mRNA in WT and *mot1-aid* cells treated with 1 mM auxin for 4 h. Values were normalised to *SCR1* mRNA levels. Average and SEM of independent experiments are shown (n = 3). Statistical analyses were performed using a one-way ANOVA and no significant differences were retrieved.
(PDF)

S5 Fig R-loop analysis in *mot1-aid* degron cells. DRIP using the S9.6 antibody at the *GCN4*, *PDC1* and *SPF1* genes in asynchronous (A), G1 synchronised (B) and S-phase enriched (C) WT and *mot1-aid* cultures treated with 1 mM auxin for 2 h. Amplicon positions are shown (top). Samples were treated *in vitro* with RNase H (green, + RNH) or not (pink, -RNH) prior to immunoprecipitation. Average and SEM of independent experiments are shown (n = 5). Statistical analyses were performed as described in the legend of Fig 1D. Only significant p-values are shown.
(PDF)

S6 Fig Replication analyses in *mot1-aid* cells overexpressing RNase H. (A) Analysis of Mot1 depletion in asynchronous, G1-synchronised and S-phase enriched *mot1-aid* cultures grown at 30ºC and treated with 1 mM auxin (NAA) for 2 h. A representative Western blot is shown. Anti-myc antibody was used to detect Mot1 aid-9myc and OsTIR1–9myc, which was used as loading control. (B) RT-qPCR measurement of *AFB1*, *NFG1* and *PDC1* mRNA in WT and *mot1-aid* cells treated with 1 mM NAA for 2 h during α-factor G1 synchronisation. Cells were transformed with pRS413 and cultured in galactose-containing medium. Values were normalised to *SCR1* mRNA levels. Average and SEM of independent experiments are shown (n = 3). Statistical analyses were performed using a one-way ANOVA and no statistically significant differences were retrieved. (C) Analysis of replication by BrdU ChIP in WT and *mot1-aid* strains at the ARS1211. Cells were transformed with pRS413 or pRS413-GALRNH1 and cultured in galactose-containing medium to induce RNase H1 expression or not. Cultures were treated with 1 mM NAA for 2 h during α-factor G1 synchronisation and then released into fresh media supplemented with 1 mM NAA. BrdU was added at timepoint 0’. A schematic representation of region analysed, and the location of the amplicon is shown (top). Relative enrichment as compared to a late-replicating region of chromosome V were calculated using the 2^-∆∆Ct^ method. Average and SEM of independent experiments are shown (n = 3 or 4). Statistical analyses were performed using a two-way ANOVA considering the interval between timepoints 10 and 30 min. Only significant p-values are shown.
(PDF)

S1 Table List of selected candidates. (XLSX)

S2 Table Recombination frequencies of selected candidates. (XLSX)

S3 Table List of temperature-sensitive mutants of the essential genes collection. (XLSX)

S4 Table Yeast strains used in this study. (PDF)

S5 Table Primers for integration used in this study. (PDF)

S6 Table Plasmids used in this study. (PDF)

S7 Table Primers for quantitative PCR used in this study. (PDF)

S1 Data Source data file. (XLSX)
