# Correction: An essential gene screening identifies yeast Mot1 as a suppressor of R-loops and genome instability

**DOI:** 10.1371/journal.pgen.1012108

**Published:** 2026-04-10

**Authors:** María E. Soler-Oliva, Rocío A. Domínguez-Sierra, Hélène Gaillard, Andrés Aguilera

There are errors in the Funding statement. The correct Funding statement is as follows: This publication is part of the project PID2022-138251NB-I00, funded by MICIU/AEI/ 10.13039/501100011033 and by ERDF/EU. This work was made with grant PID2022-138251NB-I00 funded by MICIU/AEI/10.13039/501100011033 and “ERDF/EU” as well as PID2019-104270GB-I00/BMC from the same program and grants from the European Research Council (ERC2014 AdG669898 TARLOOP), and Fundación de Investigación de la Universidad de Sevilla (FIUS22/0178) to A.A. Research was additionally supported by grant PID2022-140466NB-I00 funded by MICIU/AEI/10.13039/501100011033 and “ERDF/EU” to H.G. R.A.D-S. is recipient of a predoctoral PIF contract funded by the University of Seville (VII PPIT-US). The funders had no role in study design, data collection and analysis, decision to publish, or preparation of the manuscript.
