# Supplementary figures and images for: An essential gene screening identifies yeast Mot1 as a suppressor of R-loops and genome instability
Source: PLoS Genet. 2026 Feb 9;22(2):e1012040. doi: 10.1371/journal.pgen.1012040 (PMC12912698; doi:10.1371/journal.pgen.1012040)

# Supporting Figure S1

**A**

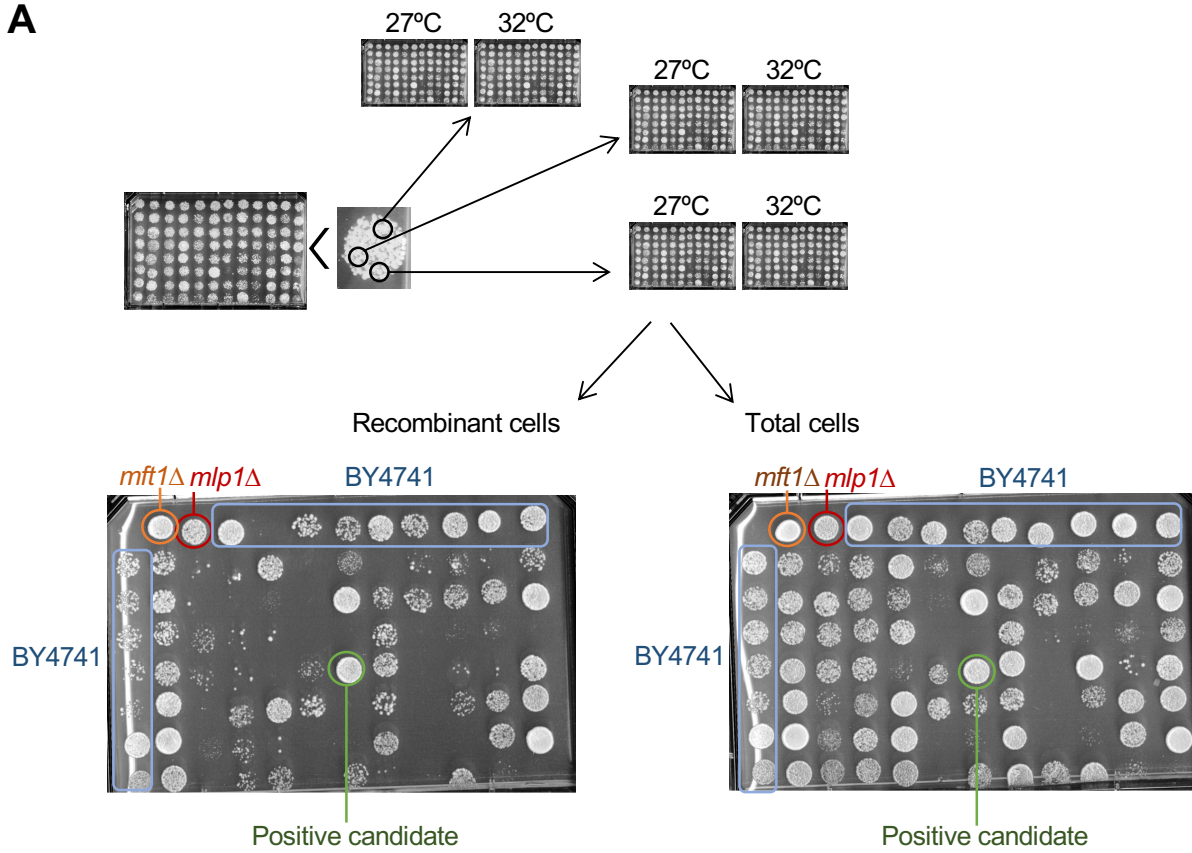

**B**

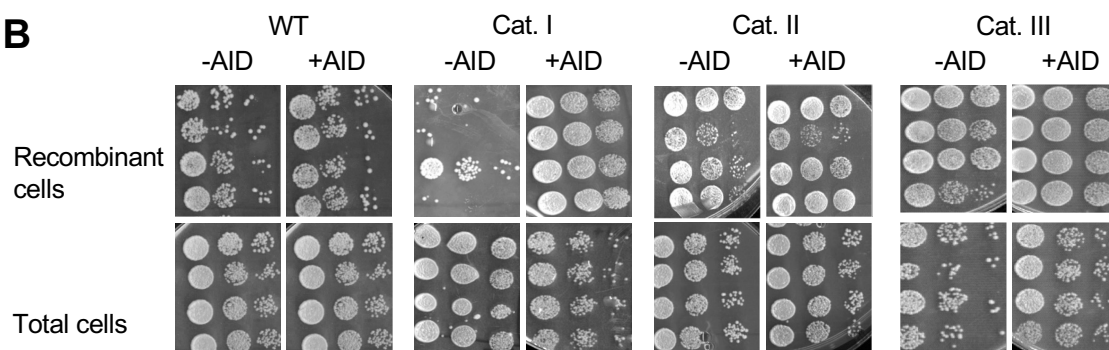

**C**

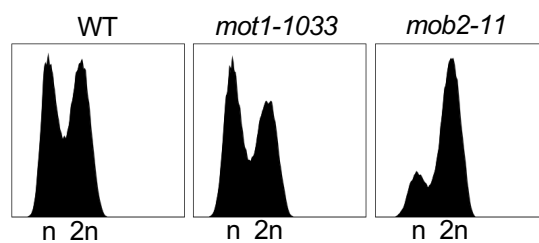

Supplement: S1 Fig — (A) Representative images of recombinant and total cells obtained from transformants of the ts collection during the first screening step. For each strain, three isolates from different areas of the transformant biomass were cultured in galactose at 27ºC and at 32ºC, as represented schematically (top). The position of WT (blue), positive controls mft1Δ (orange) and mlp1Δ (red), and an example of a positive candidate (green) are highlighted. (B) Representative images of recombinant and total cells obtained from ts collection transformants expressing AID (+AID) or not (-AID) during the second screening step. For each strain, four isolates from different areas of the transformant biomass were streaked in medium containing glucose (-AID) or galactose (+AID) and grown at 32ºC. Serial dilutions were plated on selective media to assess recombinant and total cells. Strains were classified into three categories: low level of basal recombination that increased upon AID expression (Cat. I, 42 strains), high level of basal recombination that increased upon AID expression (Cat. II, 28 strains), and high level of recombination independent of AID expression (Cat. III, 50 strains). WT and representative examples for categories I, II and III are shown (tif6-ts4, hrr25–5001 and cdc26–1, respectively). The list of candidates and their assigned categories is provided in S1 Table. (C) Flow cytometry analyses of WT, mot1–1033 and mob2–11 cells cultured at 32ºC. (PDF) [file pgen.1012040.s001.pdf]

## Supporting Figure S2

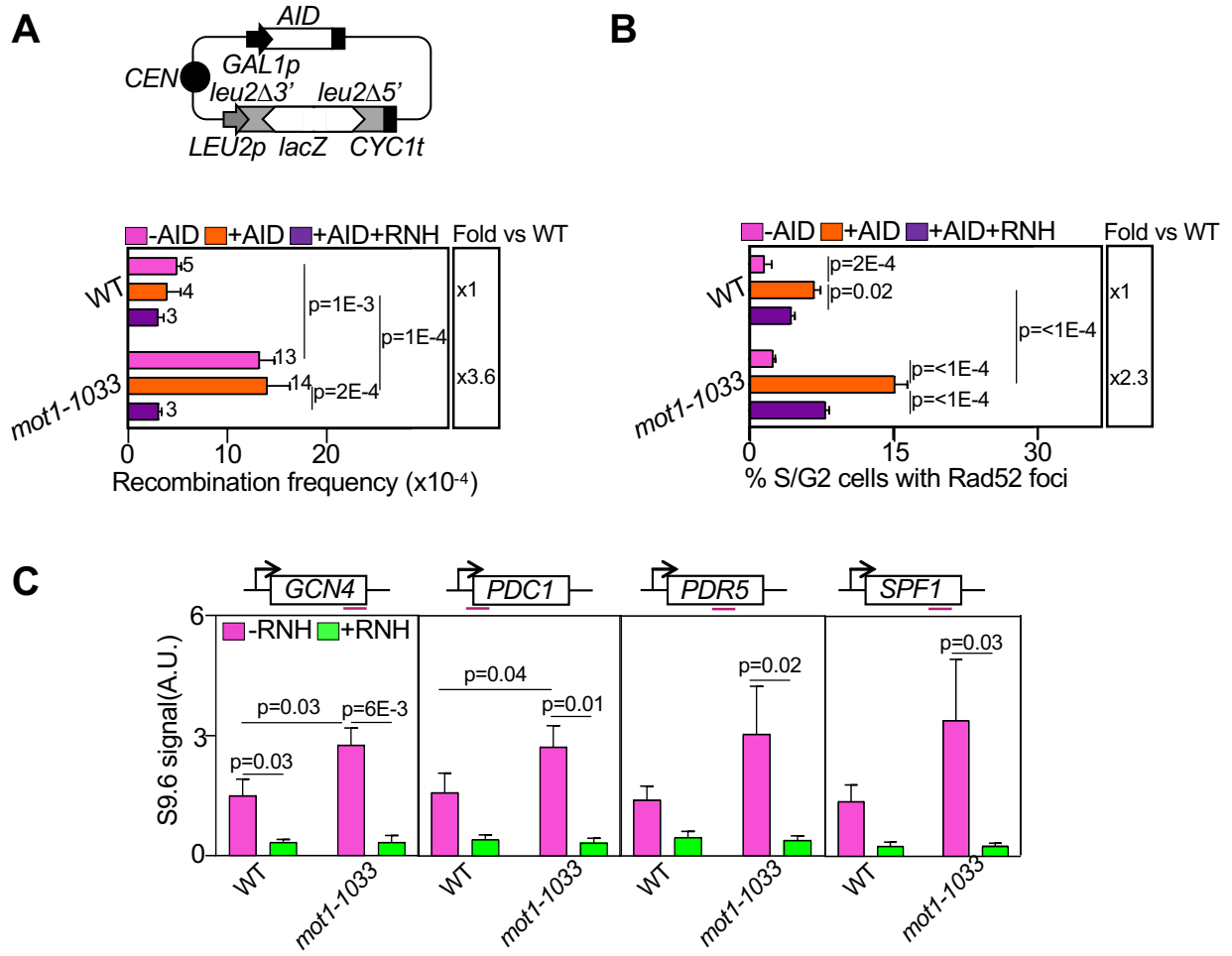

Supplement: S2 Fig — (A) Recombination analyses in WT (W303a) and mot1–1033 (mot1–1033-W303-a) cells transformed with p313LZGAID. Cells were cultured at 30ºC in glucose (no AID expression, -AID, pink) or galactose (AID expression, + AID). pRS416-GALRNH1 and pRS416 plasmids were used to overexpress RNase H1 (+RNH, purple) or not (orange). Average and SEM of fluctuation tests from six independent colonies are plotted (n = 3). Statistical analyses were performed using a two-way ANOVA followed by Holm-Šidák’s multiple comparisons test. Only significant p-values are shown. The fold increase relative to WT (+AID condition) is shown. (B) Percentage of S/G2 cells containing Rad52-YFP foci in WT and mot1–1033 cells transformed with pWJ1344 and cultured at 30ºC. Average and SEM of independent experiments in which at least 200 cells were analysed are plotted (n = 3). Details and statistical analysis as in (A). (C) DRIP using the S9.6 antibody at the GCN4, PDC1, PDR5 and SPF1 genes in WT and mot1–1033 asynchronous cultures cultured at 30ºC. Amplicon positions are shown (top). Samples were treated in vitro with RNase H (green, + RNH) or not (pink, -RNH) prior to immunoprecipitation. Average and SEM of independent experiments are shown (n = 5). Statistical analyses were performed as described in the legend of Fig 1D. Only significant p-values are shown. (PDF) [file pgen.1012040.s002.pdf]

Supporting Figure S3

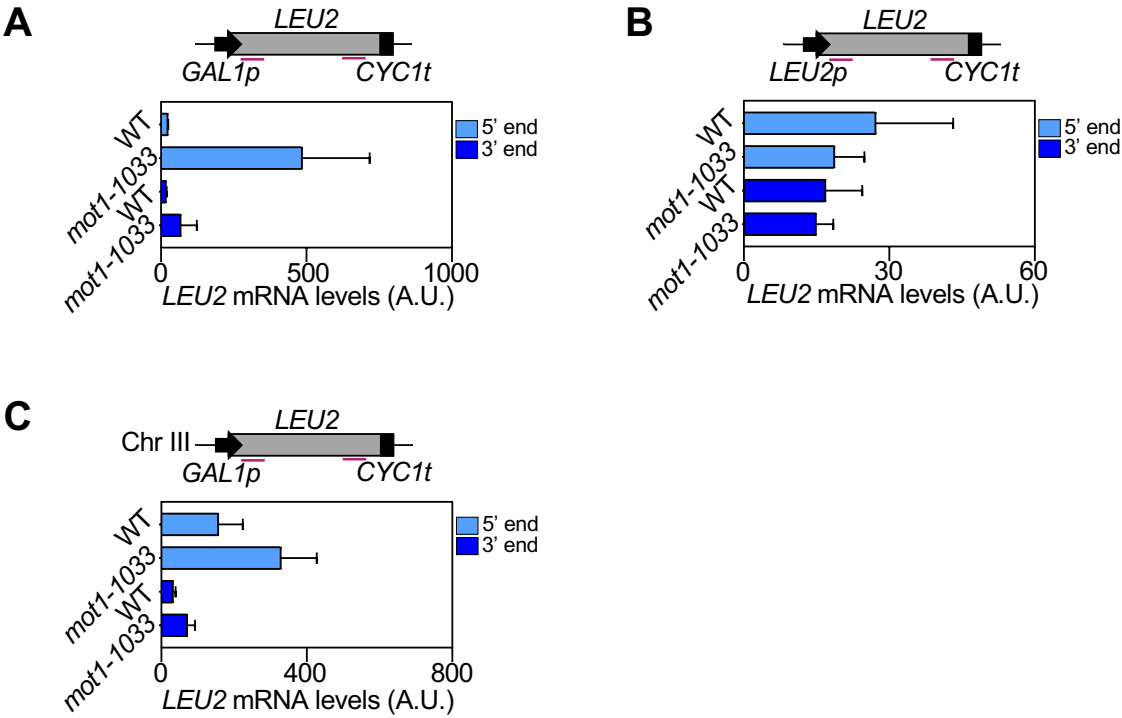

Supplement: S3 Fig — RT-qPCR measurement at the 3’ and 5’ ends of the LEU2 mRNA in WT (W303a) and mot1–1033 (mot1–1033-W303-a) strains cultured at 30ºC using recombined (A) GL-LacZ (B) L-LacZ (C) chromosomal GL-LacZ reporter systems. Values were normalized to SCR1 mRNA levels. Schematic representation of recombined reporters and amplicons are shown above each graph. Average and SEM of independent experiments are plotted (n = 3). Statistical analyses were performed using a one-way ANOVA and no statistically significant differences were retrieved. (PDF) [file pgen.1012040.s003.pdf]

Supporting Figure S4

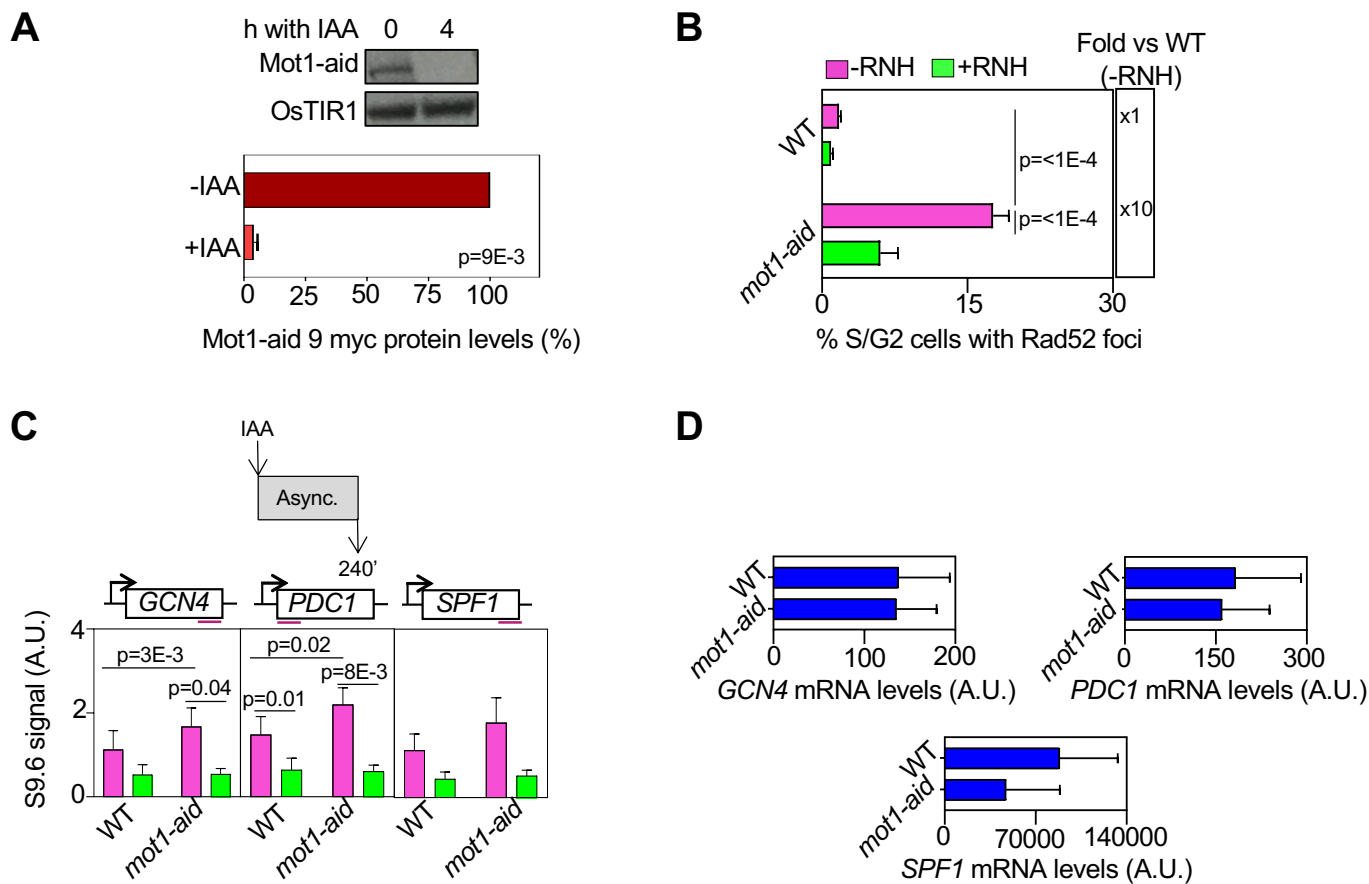

Supplement: S4 Fig — (A) Analysis of Mot1 depletion in mot1-aid cells upon auxin (IAA) treatment for 4 h. Representative Western blot analysis showing Mot1 depletion after incubation with 1 mM IAA (top). Anti-myc antibody was used to detect Mot1 aid-9myc and OsTIR1–9myc, which was used as loading control. Quantification of Mot1 protein levels in cells treated with auxin (+ IAA, light red) or not (- IAA, dark red) (bottom). Average and SEM of independent experiments are plotted (n = 3). Statistical analysis was performed using a two-tailed paired Student t-test, p-value is shown. (B) Percentage of S/G2 cells containing Rad52-YFP foci in WT and mot1-aid strains after 4 h of treatment with 1 mM auxin. pRS413-GALRNH and pRS413 plasmids were used to express RNase H1 (+RNH, green) or not (-RNH, pink). Average and SEM of independent experiments in which at least 200 cells were analysed are plotted (n = 4). Statistical analyses were performed using a two-way ANOVA followed by Holm-Šidák’s multiple comparisons test. Only significant p-values are shown. (C) DRIP using the S9.6 antibody in asynchronous WT and mot1-aid cultures treated with 1 mM auxin for 4 h. The signal obtained at the GCN4, PDC1 and SPF1 genes is plotted. Amplicon positions are shown (top). Samples were treated in vitro with RNase H (green, + RNH) or not (pink, -RNH) prior to immunoprecipitation. Average and SEM of independent experiments are shown (n = 5). Statistical analyses were performed as described in the legend of Fig 1D. Only significant p-values are shown. (D) RT-qPCR measurement of GCN4, PDC1 and SPF1 mRNA in WT and mot1-aid cells treated with 1 mM auxin for 4 h. Values were normalised to SCR1 mRNA levels. Average and SEM of independent experiments are shown (n = 3). Statistical analyses were performed using a one-way ANOVA and no significant differences were retrieved. (PDF) [file pgen.1012040.s004.pdf]

Supporting Figure S5

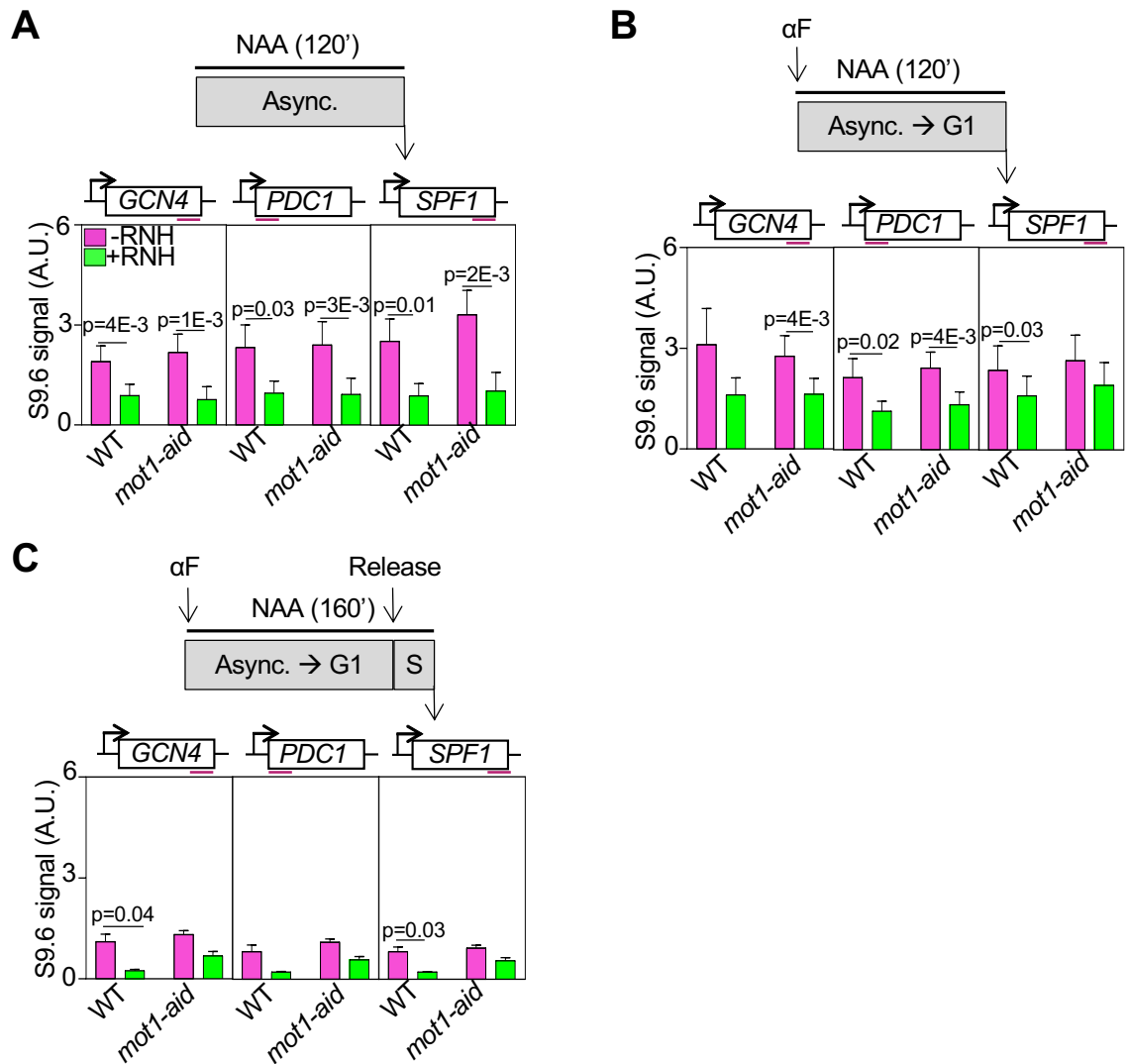

Supplement: S5 Fig — DRIP using the S9.6 antibody at the GCN4, PDC1 and SPF1 genes in asynchronous (A), G1 synchronised (B) and S-phase enriched (C) WT and mot1-aid cultures treated with 1 mM auxin for 2 h. Amplicon positions are shown (top). Samples were treated in vitro with RNase H (green, + RNH) or not (pink, -RNH) prior to immunoprecipitation. Average and SEM of independent experiments are shown (n = 5). Statistical analyses were performed as described in the legend of Fig 1D. Only significant p-values are shown. (PDF) [file pgen.1012040.s005.pdf]

## Supporting Figure S6

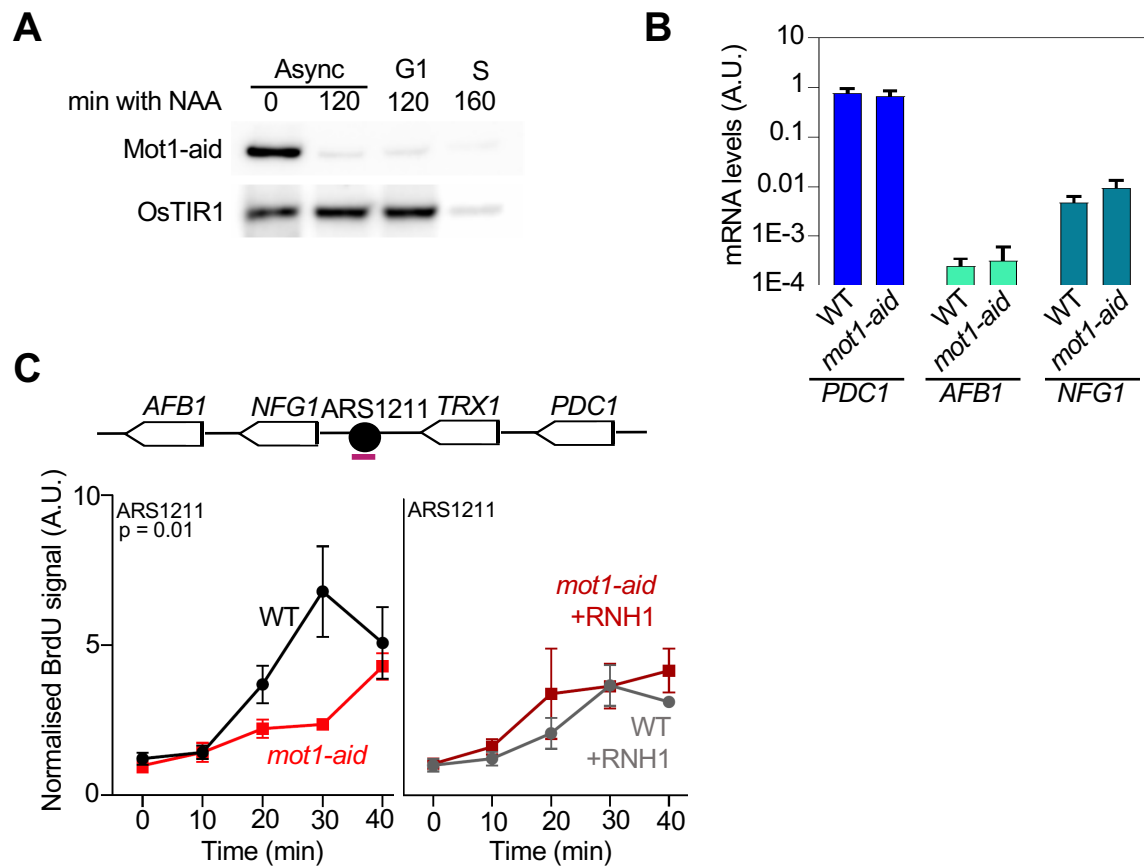

Supplement: S6 Fig — (A) Analysis of Mot1 depletion in asynchronous, G1-synchronised and S-phase enriched mot1-aid cultures grown at 30ºC and treated with 1 mM auxin (NAA) for 2 h. A representative Western blot is shown. Anti-myc antibody was used to detect Mot1 aid-9myc and OsTIR1–9myc, which was used as loading control. (B) RT-qPCR measurement of AFB1, NFG1 and PDC1 mRNA in WT and mot1-aid cells treated with 1 mM NAA for 2 h during α-factor G1 synchronisation. Cells were transformed with pRS413 and cultured in galactose-containing medium. Values were normalised to SCR1 mRNA levels. Average and SEM of independent experiments are shown (n = 3). Statistical analyses were performed using a one-way ANOVA and no statistically significant differences were retrieved. (C) Analysis of replication by BrdU ChIP in WT and mot1-aid strains at the ARS1211. Cells were transformed with pRS413 or pRS413-GALRNH1 and cultured in galactose-containing medium to induce RNase H1 expression or not. Cultures were treated with 1 mM NAA for 2 h during α-factor G1 synchronisation and then released into fresh media supplemented with 1 mM NAA. BrdU was added at timepoint 0’. A schematic representation of region analysed, and the location of the amplicon is shown (top). Relative enrichment as compared to a late-replicating region of chromosome V were calculated using the 2-∆∆Ct method. Average and SEM of independent experiments are shown (n = 3 or 4). Statistical analyses were performed using a two-way ANOVA considering the interval between timepoints 10 and 30 min. Only significant p-values are shown. (PDF) [file pgen.1012040.s006.pdf]
