# Supplementary material for: An essential gene screening identifies yeast Mot1 as a suppressor of R-loops and genome instability
Source: PLoS Genet. 2026 Feb 9;22(2):e1012040. doi: 10.1371/journal.pgen.1012040 (PMC12912698; doi:10.1371/journal.pgen.1012040)
Supplement: S4 Table — (PDF) [file pgen.1012040.s010.pdf]

Supporting Table S4. Yeast strains used in this study

Strain	Genotype	Source
Ybp249	*MATa ade2-1 can1-100 his3-11,15 leu2-3,112 trp1-1 ura3-1 bar1Δ RAD5*	B. Pardo
Ybp250	*MATα ade2-1 can1-100 his3-11,15 leu2-3,112 trp1-1 ura3-1 bar1Δ RAD5*	B. Pardo
WT-1DH11	*MATa his3Δ1 leu2Δ0 met15Δ0 ura10Δ0::kanMX4*	Li et al., 2011
mot1-1033-BY	*MATa his3Δ1 leu2Δ0 met15Δ0 ura3Δ0 mot1-1033::kanMX4*	Li et al., 2011
mob2-11-BY	*MATa his3Δ1 leu2Δ0 met15Δ0 ura3Δ0 mob2-11::kanMX4*	Li et al., 2011
mot1-1033-W303-a	*MATa leu2-3,112 trp1-1 can1-100 ura3-1 ade2-1 his3-11,15 RAD5 bar1Δ mot1-1033::kanMX4*	This study
HPBAR1-R	*MATa leu2-3,112 trp1-1 can1-100 ura3-1 ade2-1 his3-11,15 RAD5 bar1Δ hpr1Δ::HIS3*	San Martin-Alonso et al., 2021
mot1-1033-hpr1delta-W303-a	*MATa leu2-3,112 trp1-1 can1-100 ura3-1 ade2-1 his3-11,15 RAD5 bar1Δ mot1-1033::kanMX4 hpr1Δ::HIS3*	This study
SEN1-R	*MATa leu2-3,112 trp1-1 can1-100 ura3-1 ade2-1 his3-11,15 RAD5 bar1Δ sen1-1*	San Martin-Alonso et al., 2021
mot1-1033-sen1-1-W303-a	*MATa leu2-3,112 trp1-1 can1-100 ura3-1 ade2-1 his3-11,15 RAD5 bar1Δ mot1-1033::kanMX4 sen1-1*	This study
WGLZN	*MATa ade2-1 can1-100 his3-11,15 trp1-1 ura3-1 RAD5 leu2-3,112::GL-lacZ::NATMX*	Lafuente-Barquero et al., 2020
WGLZN-mot1-1033	*MATa ade2-1 can1-100 his3-11,15 trp1-1 ura3-1 RAD5 leu2-3,112::GL-lacZ::NATMX mot1-1033::kanMX4*	This study
YNK54	*MATa leu2-3,112 trp1-1 can1-100 ura3-1 ade2-1 his3-11,15 ura3-1::P_{ADH1}-OsTIR1-9Myc(URA3)*	Nishimura & Kanemaki, 2014
WT-dg-a	*MATa leu2-3,112 trp1-1 can1-100 ura3-1 ade2-1 his3-11,15 bar1Δ ura3-1::P_{ADH1}-OsTIR1-9Myc(URA3)*	This study
mot1-dg-a	*MATa leu2-3,112 trp1-1 can1-100 ura3-1 ade2-1 his3-11,15 bar1Δ ura3-1::P_{ADH1}-OsTIR1-9Myc(URA3) Mot1-AID-9myc::hphNT1*	This study
WT-LEU2-BrdUinc	*MATa leu2-3,112 trp1-1 can1-100 ura3-1 ade2-1 his3-11,15 bar1Δ ura3-1::P_{ADH1}-OsTIR1-9Myc(URA3) LEU2::BrdU–Inc*	This study
mot1-dg-LEU2-BrdUinc	*MATa leu2-3,112 trp1-1 can1-100 ura3-1 ade2-1 his3-11,15 bar1Δ ura3-1::ADH1-OsTIR1-9Myc(URA3) Mot1-AID-9myc::hphNT1 LEU2::BrdU–Inc*	This study
