# Supplementary material for: An essential gene screening identifies yeast Mot1 as a suppressor of R-loops and genome instability
Source: PLoS Genet. 2026 Feb 9;22(2):e1012040. doi: 10.1371/journal.pgen.1012040 (PMC12912698; doi:10.1371/journal.pgen.1012040)
Supplement: S5 Table — (PDF) [file pgen.1012040.s011.pdf]

Supporting Table S5. Primers for integration used in this study.

Primer	Sequence	Use
MOT1_TAG_FWD	TACGAGGAGGAGTATAATTTAGACACCTTCATCAAAACTTTACGACGTACGCTGCAGGTCGAC	Generate *mot1-aid* degron strain
MOT1_TAG_REV	ACAAAAATGACCTTGTATACGCGTCATTCCAATGCAAGAATTTGTATCGATGAATTCGAGCTCG	
MOT1_CHECK_FWD	GCTTGATCTCTTCGACCCC	Check *mot1-aid* degron strain
MOT1_CHECK_REV	ACAAAAATGACCTTGTATACGCG	
SEN1_1_CHECK_FWD	CGTTGTATTATGGTTGGTGATCC	Check *sen1-1* mutation
SEN1_1_CHECK_REV	GCAAGACAACTTCTATCTTTTGC	
5’LEU2_BRDU_CHECK	GCAGATTCCCTTTTATGGATTCC	Check integration of BrdU-Inc cassette
3’LEU2_BRDU_CHECK	GGTAGATTTAGTACTGAAGAGGAGGTCG	
RSI_BRDU_CHECK	TGAAAACCTCTGACACATGCAG	
RSII_BRDU_CHECK	CTTGATTAGGGTGATGGTTCACG	
