# Supplementary material for: An essential gene screening identifies yeast Mot1 as a suppressor of R-loops and genome instability
Source: PLoS Genet. 2026 Feb 9;22(2):e1012040. doi: 10.1371/journal.pgen.1012040 (PMC12912698; doi:10.1371/journal.pgen.1012040)
Supplement: S6 Table — (PDF) [file pgen.1012040.s012.pdf]

Supporting Table S6. Plasmids used in this study.

Plasmid	Description	Source
P313LZGAID	YCp containing the L-lacZ system under the *LEU2* promoter and the human *AID* gene under the *GAL1* promoter (*HIS3* marker)	García-Benítez et al., 2017
pWJ1344	YCp containing the *RAD52::YFP* fusion (*LEU2* marker)	Lisby et al., 2001
pSCH204	YCp prs314-LB containing the L-LacZ recombination system under the *LEU* promoter with the 3 kb fragment BamHI from LacZ inserted between the leu2 direct repeats (*TRP* marker)	Chavez & Aguilera, 1997
pRS314-GLlacZ	YCp construced by replacing the 1.22-kb *Sac*I-*Cla*I *LEU2* promoter fragment for the 0.62-kb *Sac*I-*Cla*I *GAL1* promoter fragment in pSCH204 plasmid (*TRP* marker)	Piruat & Aguilera, 1998
pARSGLB-IN	YCp containing the L-LacZ recombination system under the *GAL1* promoter cloned inward of the *ARSH4* sequence with respect to the direction of transcription (*TRP* and *URA3* markers)	Prado & Aguilera, 2005
pARSGLlacZ-IN	YCp constructed by inserting a 3-kb *BamH*I lacZ fragment at the *Bgl*II site located in between the *leu2* repeats of pARSGLB-IN plasmid (*TRP* and *URA3* markers)	Wellinger et al., 2006
pRS413	YCp containing *HIS3* as marker.	Sikorski & Hieter, 1989
pRS413-GALRNH1	YCp containing the *RNH1* gene under the *GAL1* promoter *(HIS3* marker)	García-Benítez et al., 2017
pRS416	YCp containing *URA3* as marker.	Sikorski & Hieter, 1989
pRS416-GALRNH1	YCp containing the *RNH1* gene under the *GAL1* promoter (*URA3* marker)	Huertas & Aguilera, 2003
pRS317	YCp containing *LYS2* as marker	Eriksson et al, 2004
pRS317-GALRNH1	YCp containing the *RNH1* gene under the *GAL1* promoter (*LYS2* marker)	García-Pichardo et al, 2017
pRS413-GALAID	YCp containing the human *AID* gene under the *GAL1* promoter (*URA3* marker)	Gómez-González & Aguilera, 2007
pHyg-AID*-9myc	pSM409 vector containing AID tag with the selection marker hphNT1 and the extension 9myc used to generate degron strains.	Morawska & Ulrich, 2013
p405–BrdU–Inc	*HSV–TK* gene under the control of the strong constitutive *GPD* promoter, and *hENT1* under the control of the *ADH1* promoter placed in a back- to-back orientation in the pRS315 plasmid (*LEU2* marker)	Viggiani & Aparicio, 2006
