# Supplementary material for: An essential gene screening identifies yeast Mot1 as a suppressor of R-loops and genome instability
Source: PLoS Genet. 2026 Feb 9;22(2):e1012040. doi: 10.1371/journal.pgen.1012040 (PMC12912698; doi:10.1371/journal.pgen.1012040)
Supplement: S7 Table — (PDF) [file pgen.1012040.s013.pdf]

Supporting Table S7. Primers for quantitative PCR used in this study.

Primer	Sequence	Use
GCN4_FWD	TTGTGCCCGAATCCAGTGA	ChIP/DRIP/expression levels of *GCN4*
GCN4_REV	TGGCGGCTTCAGTGTTTCTA	
PDC1_FWD	TTAACACCGTTTTCGGTTTGC	ChIP/DRIP/expression levels of *PDC1*, BrdU ChIP 2502 bp downstream of *ARS1211* (HO)
PDC1_REV	CGTTCAATTCGTTGGCGTTAC	
PDR5_FWD	GTCAGAGGCTATATTTCACTGGAGAA	ChIP/DRIP/expression levels of *PDR5*
PDR5_REV	TACGTCTTGTTTCGGCCTTAATC	
SPF1_FWD	CCCGTGGTAAACCTTTAGAAA	DRIP/expression levels of *SPF1*
SPF1_REV	ATATGAACGGCAAATTGAGAC	
LEU2_5'_FWD	GCCCCTAAGAAGATCGTCGTTT	DRIP/expression levels 5' region of *LEU2*
LEU2_5'_REV	TGGAACGAACATCAGAAATAGCTT	
LEU2_3'_FWD	CCGCATTTGGTTTGTACGAA	DRIP/expression levels 3' region of *LEU2*
LEU2_3'_REV	AGTGGCGATAGGGTTGACCTT	
LACZ_FWD	GCGCCGTGGCCTGAT	DRIP/expression levels of *LacZ* sequence
LACZ_REV	GTGCAGCGCGATCGTAATC	
AFB1_FWD	TCCGCCACCACCATATACCT	expression levels of *AFB1*
AFB1_REV	TCGCTGTCGAGAAAGGAACA	
NFG1_FWD	CCAAGCACCACCTCTTCCAGCA	expression levels of *NFG1*
NFG1_REV	AGGCAATGGCGTGAACTGGGT	
SCR1_FWD	CGCACCGTGCCCTGTT	Expression levels of *SCR1*
SCR1_REV	AGCTCTGCCCAGGACAAATTT	
2312_FWD	GGACCTCCCATGGAATTAAATTG	BrdU ChIP 2312 bp upstream of *ARS1211* (CD)
2312_REV	TGCCCATGCCAAAAGCTTAT	
ARS1211-A	CGGCTTACCGGTCTTGAAAAT	BrdU ChIP at *ARS1211*
ARS1211-B	GGAATACTTTTGCTTGAGTTGTTTAGTTT	
NR_FWD	TTCCCCACGGAAAGTTGTATCT	BrdU ChIP late replicative control region (chromosome V)
NR_REV	TGCCTGCACGCCATTGT	
